# ActiveInspect: GRPO-Optimized Multi-Sensor Evidence Selection for Industrial Defect Detection

**DOI:** 10.3390/s26154932

**Published:** 2026-08-04

**Authors:** Jingyuan Wang, Ming Wu

**Affiliations:** 1 School of Computer Science and Technology, Wuhan University of Science and Technology, Wuhan 430065, China; 202313201092@wust.edu.cn; 2 Department of Mechanical Engineering, KU Leuven and Member Flanders Make, 3001 Leuven, Belgium

**Keywords:** industrial defect detection, active visual inspection, multi-sensor fusion, vision–language model, group relative policy optimization, active sensing, multi-view inspection, 3D anomaly detection

## Abstract

Automated visual inspection is a cornerstone of modern manufacturing quality assurance, yet the effectiveness of any detection system is fundamentally bounded by the informativeness of the observations it receives. Most vision–language model (VLM) and reinforcement learning methods for industrial defect detection assume a fixed set of observations and optimize only the reasoning applied to them. We introduce ActiveInspect, which formulates inspection as budget-constrained sequential selection of multi-view, multi-modal evidence. Starting from a pre-acquired observation pool, a single policy selects an additional view or modality, zooms into a candidate region, retrieves a matched normal reference, or terminates with a verdict. The policy is initialized by perception-activated supervised fine-tuning (PA-SFT) and subsequently optimized by group relative policy optimization (GRPO) using inspection-specific rewards. Depth and point-cloud measurements are converted into VLM-compatible geometric renderings, while a structured memory integrates evidence across inspection steps. Evaluation on Real-IAD D^3^, Real-IAD, MVTec 3D-AD, MVTec-AD, VisA, and MMAD demonstrates a consistent improvement in the accuracy–observation trade-off. On Real-IAD D^3^, ActiveInspect increases image-level area under the receiver operating characteristic curve (I-AUROC) from 0.890 to 0.906 (mean over three training seeds; p<0.01) relative to the passive D^3^M baseline while reducing the average observation count from 3.0 to 2.7. It reaches 99.8% of the I-AUROC obtained by exhaustive evaluation of all 15 observations while using 18% of that observation count, and it reduces per-sample inference time by a factor of 5.3 relative to the exhaustive scan. The largest gains occur for geometry-dependent defects, including dents, warping, and concavities.

## 1. Introduction

Reliable visual inspection is essential for quality control in manufacturing because defects missed in an early stage may spread through the downstream assembly and contribute to product failure or safety-related recalls [1]. Industrial anomaly detection has advanced through reconstruction-based methods that score deviations from a learned reconstruction of normal appearance [2], embedding-based methods such as PatchCore [3] (nearest-neighbor scoring against a coreset of normal patch features) and SimpleNet [4] (a simplified discriminative pipeline for efficient deployment), and zero-/few-shot approaches such as WinCLIP [5] (window-based compositional prompting), AnomalyCLIP [6] (object-agnostic prompt learning), and PromptAD [7] (pseudo-anomaly prompts derived from normal samples). Despite their strong benchmark performance, most of these systems evaluate a single image acquired from a fixed point of view and modality. They typically return an anomaly score or map with limited ability to explain the defect or identify which additional observation would resolve an ambiguous case.

Vision–language models (VLMs) extend industrial inspection beyond anomaly scoring by supporting semantic descriptions and diagnostic reasoning, as illustrated by AnomalyGPT [8] and Myriad [9]. The MMAD benchmark [10] evaluates VLMs in seven industrial subtasks and reports an average precision of 74.9% for GPT-4o; evidence integration remains the most difficult subtask in the models evaluated. Recent reinforcement learning (RL) methods further improve VLM-based inspection: AnomalyR1 [11] applies GRPO in MMAD, IAD-R1 [12] combines PA-SFT with SC-GRPO (structured control GRPO), and AgentIAD [13] trains a VLM to invoke digital inspection tools. These methods improve reasoning over the available input, but the observation set itself remains fixed. Even AgentIAD operates within a single captured image and therefore cannot select evidence from another viewpoint or sensing modality.

A fixed observation set is restrictive when the visibility of the defect depends on the sensing mechanism. A dent or shallow concavity may have weak contrast in top-down RGB yet produce a distinct response in a surface-normal or curvature rendering. Cropping can enlarge a recorded region, but it cannot recover geometric information that is absent from the original image. Inspection therefore requires not only a decision rule but also a policy for choosing informative evidence. Active visual perception offers partial answers, along two lines. Within a single recorded image, V* [14] performs large language model (LLM)-guided visual search for small targets, Chain-of-Spot [15] interactively re-attends to regions of interest, ZoomEye [16] explores a zoom tree without additional training, and VLM-R^3^ [17] and Pixel Reasoner [18] learn cropping and zooming as reinforced actions. For physical viewpoints, SUGARL [19] studies active vision under limited observability, SeeNav-Agent [20] combines visual prompts with step-level GRPO for navigation, and NavGRPO [21] improves robustness through trajectory-diverse group comparison; this line continues a long tradition of active perception in robotics [22]. Neither class directly addresses industrial inspection, in which useful evidence may depend jointly on point of view, sensor modality, and defect type. To our knowledge, this combination has not previously been formulated as a unified learned policy over a pre-acquired multi-sensor observation pool.

We therefore introduce ActiveInspect, which presents defect detection as sequential evidence selection under an observation budget. At each step, a GRPO-optimized policy selects a view or modality, zooms into a candidate region, retrieves a normal reference, or terminates with a verdict. Geometric measurements are converted into VLM-compatible renderings, perception-activated supervised fine-tuning (PA-SFT) provides a stable initialization for GRPO, and a structured memory organizes evidence across views and modalities before final prediction. The main contributions are as follows:

1. We formulate industrial defect detection as budget-constrained sequential selection of multi-view and multi-modal evidence from a pre-acquired observation pool, unifying digital refinement and view/modality selection within a single inspection policy.
2. We define a unified action space that encompasses view/modality selection, zoom-crop, normal-reference comparison, and learned termination, complemented by VLM-compatible geometric representations and a structured cross-view evidence memory.
3. We develop a two-stage PA-SFT + GRPO training procedure with inspection-specific rewards, allowing the policy to acquire observation-selection behavior without requiring action-level annotations at every RL step.
4. A comprehensive evaluation is conducted on six benchmarks that span multi-sensor, multi-view, and single-image settings. On Real-IAD D^3^, the proposed framework reaches 99.8% of the exhaustive-scan I-AUROC while using 18% of the exhaustive observation count, with the largest gains observed in geometry-dependent defects. Systematic ablation, sensitivity, robustness, and efficiency analyses are provided to quantify individual contributions of the training, action, memory, and modality components.

## 2. Related Work

### 2.1. Industrial Anomaly Detection: From Single-View to Multi-Sensor

Industrial anomaly detection is commonly formulated as one-class learning from normal samples because real defects are sparse and difficult to enumerate. Reconstruction-based methods use the reconstruction error as an anomaly signal; DRAEM [2], for example, jointly trains reconstructive and discriminative subnets on synthetic anomalies, making performance dependent in part on the fidelity and coverage of the simulated defects. Embedding-based methods instead compare test features with normal reference statistics. PatchCore [3] performs nearest-neighbor scoring against a coreset of pre-trained patch features, whereas SimpleNet [4] and EasyNet [23] simplify this general pipeline for efficient deployment. Zero- and few-shot methods reduce dataset-specific training by exploiting vision–language pre-training: WinCLIP [5] combines compositional prompts with multi-scale features from contrastive language–image pre-training (CLIP), AnomalyCLIP [6] learns object-agnostic prompts, PromptAD [7] derives pseudo-anomaly prompts from normal samples, and UniVAD [24] provides a training-free few-shot formulation. More recent work builds directly on visual foundation models: AdaCLIP [25] adapts CLIP with hybrid learnable prompts for zero-shot detection, InCTRL [26] learns in-context residuals from few-shot normal prompts to obtain a generalist detector, AnomalyDINO [27] shows that patch-level DINOv2 features enable strong training-free few-shot detection, Dinomaly [28] demonstrates that a minimalist reconstruction framework on frozen foundation features closes the gap between multi-class and class-separated settings, and Anomaly-OV [29] couples zero-shot detection with language-based anomaly reasoning through instruction tuning. These approaches differ substantially in representation and supervision but generally evaluate a predetermined image rather than decide whether another observation is needed.

Multi-view and multi-sensor benchmarks broaden the available evidence. MVTec 3D-AD [30] pairs RGB images with 3D scans for 10 object categories, Real-IAD [31] provides 5 fixed RGB viewpoints across 30 categories, MulSen-AD [32] unifies RGB cameras, laser scanners, and lock-in infrared thermography over 15 products, and Real-IAD D^3^ [33] co-registers RGB, photometric-stereo measurements pseudo-3D and micrometer-precision point clouds across 20 categories. Corresponding methods include M3DM [34], which combines RGB and point-cloud features through hybrid contrastive learning and multiple memory banks; BTF [35], which demonstrates the competitiveness of classical 3D descriptors combined with RGB on MVTec 3D-AD; ISMP [36], which enriches point-cloud anomaly detection with internal spatial modality perception; MVAD [37] and Multi-Flow [38], which model cross-view information on Real-IAD; MV-UNet [39], which performs early multi-view fusion for metallic surfaces; and D^3^M [33], which fuses the three modalities in Real-IAD D^3^. These studies establish the value of complementary observations, but their observation schedules are fixed: the designated views or modalities are processed irrespective of sample difficulty. They also do not provide the semantic diagnostic reasoning associated with VLM-based inspection.

### 2.2. VLM- and RL-Based Industrial Inspection

VLM-based methods add semantic interpretation to industrial anomaly detection by generating descriptions of defect type, location, and severity. AnomalyGPT [8] uses simulated anomaly image–text pairs, a lightweight image decoder, and a prompt learner to support multi-turn diagnosis, although its supervision and localization remain tied to synthetic anomalies and text outputs. Myriad [9] injects anomaly maps from pre-trained visual experts into a multimodal model through an adapter; its diagnostic performance therefore depends on the quality of the underlying expert outputs. MMAD [10] provides a seven-subtask benchmark for this class of models and shows that comprehensive analysis requiring the integration of multiple evidence types remains particularly difficult, while text-only position descriptions offer limited localization precision.

RL has subsequently been used to improve VLM reasoning and tool use. AnomalyR1 [11] combines GRPO with a reasoned-outcome alignment reward, IAD-R1 [12] uses PA-SFT followed by SC-GRPO with four reward dimensions, and LAD-Reasoner [40] applies GRPO to logical anomaly detection on MVTec LOCO AD. AgentIAD [13] is the most closely related tool-using system: it trains a VLM to invoke a perceptive zoomer, comparative retriever, and web searcher, yielding a reported 5.92% improvement on MMAD. Nevertheless, these methods optimize reasoning or tool invocation after the input image has been captured. AgentIAD can refine or compare regions within that image, but it does not choose among alternative viewpoints or sensor modalities, and its tool calls are not defined as a unified structured observation-selection policy.

### 2.3. GRPO, Active Visual Perception, and Cost-Aware Sensing

GRPO was introduced in DeepSeekMath [41] as a critic-free alternative to proximal policy optimization (PPO), with advantages estimated from relative rewards within a sampled group. DeepSeek-R1 [42] further demonstrated the effectiveness of GRPO with verifiable rewards for reasoning tasks. Visual extensions include Visual-RFT [43], which uses IoU and accuracy rewards; Vision-R1 [44], which studies multimodal mathematical reasoning; and R1-VL [45], which introduces step-wise dense rewards. More recent methods incorporate perception operations into the reasoning trajectory. VLM-R^3^ [17] allows a model to localize, crop, or request enhanced evidence; Pixel Reasoner [18] treats zooming and frame selection as explicit actions; and ViGoRL [46] links reasoning steps to visual coordinates through iterative zoom feedback. These studies motivate three choices in our training design: a supervised fine-tuning (SFT) initialization before RL [12,17,44], step-level reward components [20,45], and an explicit comparison of Kullback–Leibler (KL) regularization strategies. The latter remains unsettled: the original GRPO formulation and AnomalyR1 retain KL regularization, DAPO [47] removes it, and PAPO [48] introduces a perception-aware variant.

Active visual perception can also be separated by the source of additional information. Single-image methods operate within the recorded pixel space: V* [14] performs LLM-guided visual search, Chain-of-Spot [15] focuses interactively on regions of interest, and ZoomEye [16] uses training-free tree-based zooming; VLM-R^3^ and Pixel Reasoner learn related operations. By contrast, viewpoint-selection methods seek observations from different sensor poses. SUGARL [19] studies active vision under partial observability, SeeNav-Agent [20] combines dual-view prompts with step-level GRPO, NavGRPO [21] uses trajectory-diverse group comparison, and GROVE [49] learns open-vocabulary physical skills from VLM-derived rewards. The former class cannot recover cues that were not recorded in the initial image, whereas the latter primarily addresses navigation at scene scale. Industrial defect inspection requires fine-grained selection across both views and modalities; the present work studies this problem in a controlled pre-acquired observation pool.

Budget-aware observation selection also has a long history outside VLMs, in active perception, metrology, and robotics. Bajcsy et al. [22] frame active perception as intervening in the acquisition process itself rather than passively processing given data. Scott et al. [50] survey view planning for automated three-dimensional object reconstruction and inspection, where sensing positions are selected under explicit acquisition-cost models, and Zeng et al. [51] review next-best-view planning in robot active vision. On the decision-theoretic side, Krause and Guestrin [52] select near-optimal observation subsets under budget constraints by exploiting submodularity, and Satsangi et al. [53] scale active perception in partially observable Markov decision processes through submodular value functions. These formulations optimize geometric coverage or information-theoretic objectives with hand-specified sensor models, whereas ActiveInspect learns the acquisition value of views and modalities end-to-end from task reward within a VLM policy. The two lines are complementary: explicit per-action cost models from this literature are a natural refinement of our uniform observation count (Section 6).

### 2.4. Summary and Positioning

Table 1 positions representative methods according to the capabilities relevant to this study. Conventional single- and multi-view detectors process fixed observation sets and do not make explicit evidence-selection decisions. VLM-RL methods add semantic reasoning and, in some cases, digital tools, but generally remain restricted to the initially supplied image. ActiveInspect combines structured VLM reasoning, RL-based policy optimization, digital refinement, selection of views and modalities from a pre-acquired pool, and learned termination within a common evidence memory.

## 3. Methodology

### 3.1. Problem Formulation

We cast industrial defect inspection as a budget-constrained partially observable Markov decision process (POMDP) (S,A,T,Ω,R,B), where S denotes the state space, A the action space, T the deterministic transition function, Ω the finite observation space defined below, *R* the reward function, and *B* the observation budget. The observation space is the finite set of all observations that the sensor rig can produce,   

(1) $$\Omega =\left\{o_{v,m}|v\in V,\;m\in M\right\},\;|\Omega |=|V|\;|M|<\infty ,$$

where V is the finite set of viewpoints and M is the finite set of raw sensor modalities; on Real-IAD D^3^, these are RGB, photometric-stereo pseudo-3D, and 3D point cloud, so that |V||M|=5×3=15 matches the observation pool of Section 4.1. Non-RGB modalities are presented to the VLM through the geometric renderings of Section 3.4 (surface-normal, curvature, and view-dependent point-cloud projections); these renderings are alternative representations of the same underlying observation and do not enlarge Ω. After rendering, every element of Ω is a bounded image $o\in {\left[0,255\right]}^{H_{\mathrm{in}}\times W_{\mathrm{in}}\times 3}$, so Ω is a finite subset of a compact space and no additional topological structure is required. All recurring symbols are summarized in Table 2. This level of explicit algebraic specification follows the established practice of structure-based property estimation in other quantitative disciplines, where system-level properties are computed exactly from compact structural descriptors: the structure–property index of Wiener [54], the counting polynomials of Hosoya [55], and the M-polynomial of Deutsch and Klavžar [56] each derive property values directly from the formal structural definition. We adopt the same standard of explicitness for the observation space, the state, and the transition operator. For each product instance, the available observation pool is

(2) $$O=\{o_{v,m}|v\in V,\;m\in M\}\subseteq \Omega ,$$

which collects the observations actually acquired for that instance. A global scan of the default observation also produces a candidate region set $R=\{r_{1},\ldots ,r_{N}\}$ (Section 3.3); K denotes the dataset-specific defect vocabulary. At most, *B* observations may be presented to the agent.

Before formalizing the state, we define its components. The structured evidence memory $E_{t}=\left\{e_{1},\ldots ,e_{t-1}\right\}$ stores one entry per executed evidence-acquiring action, with the entry format $e_{t}$ specified in Equation (17) of Section 3.5. The set $U_{t}^{\mathrm{vm}}\subseteq V\times M$ contains the view–modality pairs not yet visited (the parameter set of action family A1), and $U_{t}^{r}\subseteq R$ contains the candidate regions not yet visited (the parameter sets of A2 and A3). After the initial observation, the state at decision step t∈{1,…,B−1} is

(3) $$s_{t}=\left(E_{t},\;U_{t}^{\mathrm{vm}},\;U_{t}^{r},\;B-1-t\right),$$

where the last component B−1−t counts the evidence acquisitions that remain possible after step *t*: the initial observation at t=0 and the acquisitions of steps 1,…,t occupy t+1 of the *B* observation slots, so the counter reaches 0 at the final decision step t=B−1. The policy $\pi_{\theta }\left(a_{t}|s_{t}\right)$ selects $a_{t}\in A$. Let

(4) param:A∖{A4}→(V×M)∪R

map each evidence-acquiring action to its argument, i.e., param(A1(v,m))=(v,m) and param(A2(r))=param(A3(r))=r, and let ∖ denote set difference. The state then updates deterministically under T, with the two candidate sets maintained separately:

(5) $$\begin{array}{cc} E_{t+1} & =E_{t}\cup \left\{e_{t}\right\},\\ U_{t+1}^{\mathrm{vm}} & =\left\{\begin{array}{cc} U_{t}^{\mathrm{vm}}\setminus \left\{\mathrm{param}\left(a_{t}\right)\right\} & \mathrm{if}\;a_{t}\in A1,\\ U_{t}^{\mathrm{vm}} & \mathrm{otherwise}, \end{array}\right.\\ U_{t+1}^{r} & =\left\{\begin{array}{cc} U_{t}^{r}\setminus \left\{\mathrm{param}\left(a_{t}\right)\right\} & \mathrm{if}\;a_{t}\in A2\cup A3,\\ U_{t}^{r} & \mathrm{otherwise}. \end{array}\right. \end{array}$$

The episode ends when $a_{t}=A4$ or the budget is exhausted after step t=B−1. A trajectory is the ordered, alternating sequence of states and actions

(6) $$\tau =(s_{1},a_{1},s_{2},a_{2},\ldots ,s_{T},a_{T})$$

generated by rolling out $\pi_{\theta }$ under T, beginning at the first decision step t=1; the mandatory initial observation at t=0 precedes any decision and is not part of the action sequence. The final action $a_{T}$ is the terminating event: either an explicit A4 selected by the policy at a decision step T≤B−1, or the automatic termination applied in the exhausted-budget state reached after step B−1, in which case T=B. In both cases, the trajectory contains exactly T−1 evidence-acquiring actions $a_{1},\ldots ,a_{T-1}$, so |τ|=T equals the number of observations consumed, namely the initial observation plus the T−1 acquisitions; this convention matches the observation-counting protocol of Section 4.1. The final verdict $\hat{y}\in Y$ is produced from the terminal memory $E_{T}$ (Section 3.5), $y^{*}\in Y$ denotes the ground-truth label, and $E_{\tau ∼\pi_{\theta }}\left[\cdot \right]$ averages over trajectories generated in this way. The objective trades prediction quality against trajectory length:

(7) $$\theta^{*}=\arg\max_{\theta }\;E_{\tau ∼\pi_{\theta }}\;\left[R(\tau )\right],\;R\left(\tau \right)=R_{\mathrm{task}}\left(\tau \right)-\lambda \;\left|\tau \right|,$$

where $R_{\mathrm{task}}$ collects the terminal and step-level quality terms of the trajectory and λ is the efficiency coefficient, whose sensitivity is quantified in Section 4.9. Section 3.8 specifies the terminal and step-level terms used to instantiate Equation (7).

**Table 2:** Summary of notation.

Symbol	Meaning	Symbol	Meaning
Ω	finite observation space of the rig, Equation (1)	O	per-instance observation pool, O⊆Ω
V, M	viewpoint set, modality set	$o_{v,m}$	observation at viewpoint *v*, modality *m*
R	candidate-region set, |R|≤13	K	dataset-specific defect vocabulary
*B*	observation budget	$s_{t}$	state at step *t*, Equation (3)
$E_{t}$	structured evidence memory	$e_{t}$	evidence entry, Equation (17)
$U_{t}^{\mathrm{vm}}$	unvisited view–modality pairs	$U_{t}^{r}$	unvisited candidate regions
A, A1–A4	action space and its four families	param(·)	argument of an evidence action, Equation (4)
τ, |τ|=T	trajectory; *T* = number of observations consumed, Equation (6)	$\pi_{\theta }$	policy with parameters θ
$\hat{y}$, $y^{*}$	predicted verdict, ground-truth label	Y	verdict space
R(τ)	trajectory reward, Equation (30)	λ	efficiency coefficient
$y_{t}=\left(b_{t},k_{t}\right)$	step-level hypothesis	$c_{t}$	step-level confidence
G(r)	cross-view evidence group, Equation (18)	S(x,y)	pixel-level anomaly map, Equation (21)
$A_{i}$	group-relative advantage	$\rho_{t}$	importance ratio in Equation (25)
f(·)	frozen visual encoder, Equation (10)	N, $N_{v}$	normal reference bank, viewpoint-matched subset
κ	score-map kernel tying coefficient (Section 3.5)	$N_{\mathrm{tok}}$	generated-token count of the structured output (Section 3.7)

### 3.2. Overview

**Figure 1:**
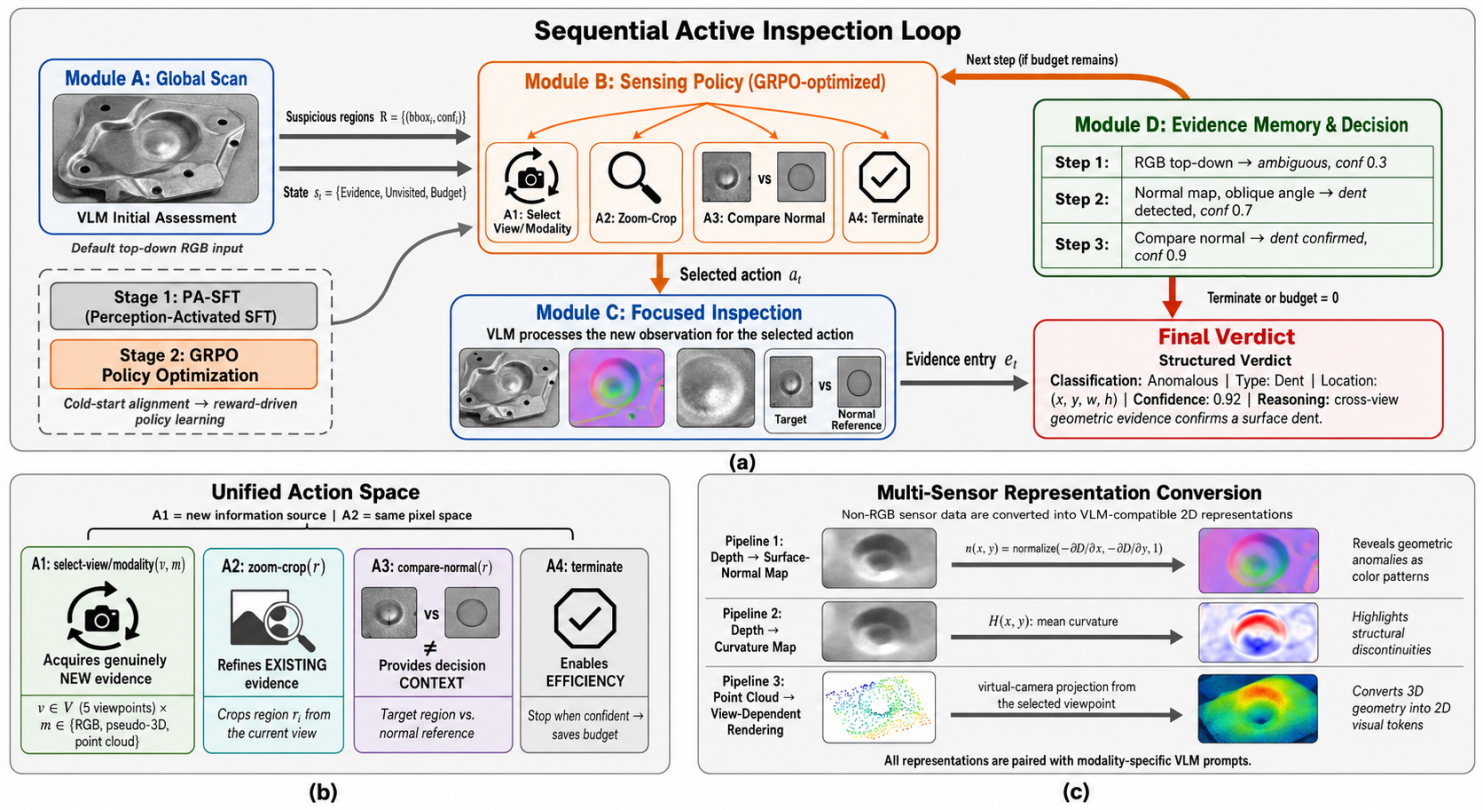
Overview of ActiveInspect. (**a**) Sequential active inspection loop with structured evidence memory and final verdict generation. (**b**) Unified action space. (**c**) Multi-sensor representation conversion: VLM-compatible renderings of the geometric measurements. In (**c**), the surface-normal rendering encodes the three components of the surface normal in the red, green, and blue channels (Equation (13)); the curvature rendering uses a diverging colormap in which blue denotes concavity, white denotes flat regions, and red denotes convexity; and the point-cloud rendering is colored by height (sequential colormap), normal direction (RGB), or curvature (diverging colormap).

Figure 1 summarizes the framework, and Algorithm 1 details the inference procedure. For each product instance, the agent selects an action, retrieves the corresponding evidence, updates the memory, and repeats until it terminates or exhausts the budget.

The three panels of Figure 1 correspond to the three pillars of the framework. Panel (a) traces one inspection episode through its four modules. Module A performs the global scan on the default top-down RGB input and produces the suspicious-region set $R=\{\left({\mathrm{bbox}}_{i},{\mathrm{conf}}_{i}\right)\}$ (bounding boxes with initial confidences) together with the state $s_{t}$ (evidence, unvisited candidates, remaining budget); the two-stage training pipeline (PA-SFT cold-start alignment followed by GRPO policy optimization) yields the sensing policy of Module B, which selects one of the four actions A1–A4; Module C (focused inspection) executes the selected action by passing the requested observation, in its rendered form, through the VLM; and the resulting evidence entry $e_{t}$ is appended to the structured memory of Module D. The worked example in Module D illustrates the intended accumulation pattern: an ambiguous top-down RGB view (confidence 0.3) is followed by an oblique surface-normal map that reveals a dent (0.7) and a normal-reference comparison that confirms it (0.9), after which the episode terminates and the structured verdict reports class, defect type, location, confidence, and a cross-view reasoning summary; control otherwise returns to Module B while budget remains. Panel (b) expands the action space along its information roles: A1 acquires genuinely new evidence by selecting an unvisited view–modality pair, A2 refines existing evidence within the same pixel space via zoom-cropping, A3 supplies decision context by juxtaposing the target region with a retrieved normal reference, and A4 terminates to convert confidence into budget savings. Panel (c) details the representation-conversion pipelines of Section 3.4: depth is differentiated into surface-normal maps (Equation (12)) and mean-curvature maps (Equation (14)), and point clouds are projected through a virtual camera into view-dependent renderings; every rendering is paired with a modality-specific interpretation prompt before entering the VLM. The interaction between the panels is unidirectional at inference time: (c) supplies the observations consumed by the loop in (a), and (b) defines the interface through which that loop acquires and integrates evidence.

Algorithm 1 relies on four VLM-implemented subroutines with the following signatures. GlobalScan$:(\pi_{\theta },o_{0})\mapsto R$ parses the initial observation and returns the candidate-region set as bounding boxes with initial confidences (|R|≤13; Section 3.3). Execute$:\left(a_{t},O,N\right)\mapsto {\tilde{o}}_{t}$ retrieves the observation requested by $a_{t}$: the pool element $o_{v,m}$ for A1 (Equation (8)), the resized crop of Equation (9) for A2, and the comparison image of Equation (10) for A3. StepAnalysis$:\left(\pi_{\theta },{\tilde{o}}_{t},E_{t}\right)\mapsto e_{t}$ produces the structured evidence entry of Equation (17). VerdictGeneration$:\left(\pi_{\theta },E_{T}\right)\mapsto \left(\hat{y},\hat{p}\right)$ returns the verdict and a verbalized anomaly probability $\hat{p}\in \left[0,1\right]$ from the serialized memory, initialized by the consensus of Equation (19). Each subroutine is a single constrained-decoding VLM call with a prompt template that is fixed across datasets.
**Algorithm 1** ActiveInspect Inference**Require:** Product observation pool O, budget *B*, policy $\pi_{\theta }$, normal reference bank N**Ensure:** Final verdict $\hat{y}$ with reasoning trace 1: $o_{0}\leftarrow$ default observation (top-down RGB)▹ Consumes 1 of *B* observations 2: R← GlobalScan$\left(\pi_{\theta },o_{0}\right)$▹ Initial hypothesis + region candidates 3: $U_{1}^{\mathrm{vm}}\leftarrow V\times M\setminus \left\{\left(v_{0},m_{0}\right)\right\}$; $U_{1}^{r}\leftarrow R$; $E_{1}\leftarrow \emptyset$ 4: **for** t=1,…,B−1 **do** 5:       $s_{t}\leftarrow \left(E_{t},\;U_{t}^{\mathrm{vm}},\;U_{t}^{r},\;B-1-t\right)$ 6:       $a_{t}∼\pi_{\theta }\left(\cdot |s_{t}\right)$ with invalid-action masking 7:       **if** $a_{t}=A4:\mathrm{terminate}$ **then** 8:             **break** 9:       **end if**10:       ${\tilde{o}}_{t}\leftarrow$ Execute$\left(a_{t},O,N\right)$▹ Acquire observation11:       $e_{t}\leftarrow$ StepAnalysis$\left(\pi_{\theta },{\tilde{o}}_{t},E_{t}\right)$▹ Generate evidence entry12:       $E_{t+1}\leftarrow E_{t}\cup \left\{e_{t}\right\}$13:       **if** $a_{t}\in A1$ **then** $U_{t+1}^{\mathrm{vm}}\leftarrow U_{t}^{\mathrm{vm}}\setminus \left\{\left(v_{t},m_{t}\right)\right\}$14:       **else if** $a_{t}\in A2\cup A3$ **then** $U_{t+1}^{r}\leftarrow U_{t}^{r}\setminus \left\{r_{t}\right\}$15:       **end if**16: **end for**17: $\hat{y}\leftarrow$ VerdictGeneration$\left(\pi_{\theta },E_{T}\right)$▹T=t on A4; T=B after budget exhaustion18: **return** $\hat{y}$

**Computational complexity.** The cost of sequential selection scales linearly with trajectory length. Each step issues one VLM forward pass whose input comprises at most two images and the serialized memory, so the per-sample inference cost is $O(T\cdot C_{\mathrm{VLM}})$ with T≤B, where $C_{\mathrm{VLM}}$ denotes the cost of a single call. Exhaustive processing instead costs $O(|O|\cdot C_{\mathrm{VLM}})$ with |O|=15 on Real-IAD D^3^. Because learned termination keeps the realized mean at T=2.7, the framework issues 5.6× fewer observations than the exhaustive scan, consistent with the measured 5.3× runtime reduction of Section 4.7.

### 3.3. Unified Inspection Action Space

The action space A=A1∪A2∪A3∪A4 comprises four action families.

**A1: select-view/modality.** This action is parameterized by $\left(v,m\right)\in U_{t}^{\mathrm{vm}}$ and retrieves $o_{v,m}$ from the observation pool. Its valid parameter set at step *t* is

(8) $$A_{1}\left(t\right)=\left\{\left(v,m\right):\left(v,m\right)\in U_{t}^{\mathrm{vm}}\right\}.$$

**A2: zoom-crop.** This action is parameterized by a region index r∈R. The global scan forms region candidates from a quantized 3×3 grid and up to four additional regions proposed by the VLM (N≤13). The selected crop $I_{r}$ is resized to the VLM input resolution $H_{\mathrm{in}}\times W_{\mathrm{in}}$:

*I_r_* = Resize(*I*[*y*_1_ : *y*_2_, *x*_1_ : *x*_2_], *H*_in_, *W*_in_), (*x*_1_, *y*_1_, *x*_2_, *y*_2_) = BBox(*r*). (9)

The 3×3 granularity implements a coarse-to-fine design: a grid cell first localizes the suspicious area, the subsequent resize to the native input resolution restores fine detail, and defects smaller than a cell are covered by the up to four free-form VLM-proposed boxes rather than by the grid itself. The effect of the grid granularity is quantified in Section 4.9.

**A3: compare-normal.** For a selected region *r*, this action retrieves the nearest normal reference from N and constructs a side-by-side comparison:

(10) $$r^{*}=\arg\min_{r^{\prime }\in N_{v}}{∥f\left(I_{r}\right)-f\left(I_{r^{\prime }}\right)∥}_{2},\;I_{\mathrm{cmp}}=Cᴏɴᴄᴀᴛ\left(I_{r},\;I_{r^{*}}\right),$$

where $f:{\left[0,255\right]}^{H_{\mathrm{in}}\times W_{\mathrm{in}}\times 3}\to R^{d}$ is the frozen visual encoder of the backbone VLM, implemented as the $l_{2}$-normalized mean-pooled visual-token embedding with d=3584, and $N_{v}$ is the viewpoint-matched subset of the normal reference bank.

**A4: terminate.** This action triggers verdict generation from the accumulated evidence $E_{T}$. It is available at every step; if the policy has not selected it by the end of step B−1, the episode terminates automatically once the budget is exhausted, and verdict generation is invoked with T=B (the forced termination of Equation (6)).

**Action masking.** Invalid actions are masked by assigning them log-probability −∞:

(11) $$\log\pi_{\theta }\left(a_{t}|s_{t}\right)\leftarrow \left\{\begin{array}{cc} \log\pi_{\theta }\left(a_{t}|s_{t}\right) & \mathrm{if}\;a_{t}\in A_{\mathrm{valid}}\left(t\right),\\ -\infty & \mathrm{otherwise}, \end{array}\right.$$

where $A_{\mathrm{valid}}\left(t\right)=A_{1}\left(t\right)\cup A_{2}\left(t\right)\cup A_{3}\left(t\right)\cup \left\{A4\right\}$, with $A_{1}\left(t\right)$ derived from $U_{t}^{\mathrm{vm}}$ and $A_{2,3}\left(t\right)$ from $U_{t}^{r}$.

### 3.4. Multi-Sensor Representation

Non-RGB measurements are rendered as VLM-compatible false-color images and paired with modality-specific prompts that describe how the encoding should be interpreted.

**Surface-normal rendering.** For a depth map D(x,y), the local surface normal is computed as

(12) $$n\left(x,y\right)=\mathrm{normalize}\;\left(-\frac{\partial D}{\partial x},\;-\frac{\partial D}{\partial y},\;1\right),$$

where partial derivatives are computed via 3×3 Sobel filters. The normal components are mapped linearly to the RGB channels:

(13) $$\left[\begin{array}{c} R(x,y)\\ G(x,y)\\ B(x,y) \end{array}\right]=\left\lfloor \frac{255}{2}\;\left(n(x,y)+1\right)\right\rfloor ,\;1={\left(1,1,1\right)}^{⊤},$$

where the affine map and the floor operator act elementwise, so each normal component in [−1,1] is encoded into {0,…,255}. For noisy depth maps, bilateral filtering ($\sigma_{s}=5$, $\sigma_{r}=0.05$) is applied before differentiation.

**Curvature rendering.** Mean curvature *H* represents second-order variation in the surface geometry:

(14) $$H\left(x,y\right)=\frac{\left(1+D_{x}^{2}\right)\;D_{yy}-2\;D_{x}D_{y}D_{xy}+\left(1+D_{y}^{2}\right)\;D_{xx}}{2\;{\left(1+D_{x}^{2}+D_{y}^{2}\right)}^{3/2}}.$$

Curvature values are clipped and normalized:

(15) $$\tilde{H}\left(x,y\right)=\mathrm{clip}\;\left(\frac{H\left(x,y\right)-\mu_{H}}{\sigma_{H}},\;-3,\;3\right),$$

where $\mu_{H}$ and $\sigma_{H}$ are per-image statistics. The normalized values are rendered with a diverging colormap (concavity → blue, flat → white, convexity → red).

**Point-cloud rendering.** Point clouds are converted into view-dependent 2D renderings using a virtual camera. Point coordinates are expressed in the dataset object frame F; for a selected viewpoint *v*, the rigid transform $\left[R_{v}|t_{v}\right]\in \mathrm{SE}\left(3\right)$ maps F to the camera frame $C_{v}$, and K is the intrinsic matrix matched to the dataset’s RGB camera. For a point $p_{i}={\left(x_{i},y_{i},z_{i}\right)}^{⊤}$ with estimated normal ${\hat{n}}_{i}$ and curvature ${\hat{H}}_{i}$, computed from its k=30 nearest neighbors, the projected pixel coordinates satisfy the homogeneous pinhole model

(16) $$z_{i}^{\left(c\right)}\left[\begin{array}{c} u_{i}\\ v_{i}\\ 1 \end{array}\right]=K\;\left[R_{v}|t_{v}\right]\left[\begin{array}{c} p_{i}\\ 1 \end{array}\right],\;z_{i}^{\left(c\right)}>0,$$

where the depth $z_{i}^{\left(c\right)}$ of $p_{i}$ in $C_{v}$ is the projective scaling factor that replaces the proportionality of a scale-free formulation. Points are splatted with a 2-pixel radius under z-buffer occlusion handling. Each point is colored by one of three schemes: height ($z_{i}\to$ sequential colormap), normal (${\hat{n}}_{i}\to$ RGB), or curvature (${\hat{H}}_{i}\to$ diverging colormap).

### 3.5. Structured Evidence Memory

Each inspection step records an evidence entry:

(17) $$e_{t}=\left(t,\;a_{t},\;v_{t},\;m_{t},\;r_{t},\;y_{t},\;c_{t},\;q_{t}\right).$$

Here, $y_{t}=\left(b_{t},k_{t}\right)$ is the step-level hypothesis, with binary state $b_{t}\in \left\{\mathrm{normal},\mathrm{anomalous}\right\}$ and defect type $k_{t}\in K\cup \left\{⌀\right\}$; $c_{t}\in \left[0,1\right]$ is the associated confidence; and $q_{t}$ is a natural-language observation. The confidence is parsed from the probability field in the VLM’s structured JSON output. It is used both to weight evidence in memory and to derive the continuous anomaly score required for threshold-independent evaluation (Section 4.1). The memory at termination is $E_{T}=\left\{e_{1},\ldots ,e_{T-1}\right\}$.

Evidence entries that refer to the same region are collected into a region group,

(18) $$G\left(r\right)=\left\{e_{i}\in E_{T}:r_{i}=r\right\},$$

and a group is termed *cross-view evidence* when its entries originate from at least two distinct view–modality pairs, i.e., $\left|\{\left(v_{i},m_{i}\right):e_{i}\in G\left(r\right)\}\right|\geq 2$. These groups expose agreements and conflicts across modalities to the verdict generator.

At termination, $E_{T}$ is serialized as a structured prompt. Let Y={normal}∪({anomalous}×K) denote the verdict space. A confidence-weighted consensus is first computed as

(19) $${\hat{y}}_{\mathrm{cons}}=\arg\max_{y\in Y}\sum_{e_{t}\in E_{T}}c_{t}\cdot 1\left[y_{t}=y\right].$$

Entries with $b_{t}=\mathrm{anomalous}$ but unspecified type $k_{t}=⌀$ match no element of Y, so the indicator excludes them from the consensus sum; their content still reaches the verdict generator through the serialized memory. The consensus initializes a final VLM pass over the serialized memory, which produces the verdict $\hat{y}$ and a verbalized anomaly probability.

**Pixel-level anomaly score map.** For pixel-level evaluation (P-AUROC and AUPRO; both metrics are defined in Section 4.1), each evidence entry $e_{t}$ with confidence $c_{t}$ and bounding box $\left(x_{1}^{t},y_{1}^{t},x_{2}^{t},y_{2}^{t}\right)$ is projected onto a continuous anomaly map $S\in {\left[0,1\right]}^{H\times W}$. A Gaussian-weighted kernel centered in the bounding box defines the step-level map

(20) $$S_{t}\left(x,y\right)=c_{t}\cdot 1\left[b_{t}=\mathrm{anomalous}\right]\cdot \exp\;\left(-\frac{{\left(x-{\bar{x}}_{t}\right)}^{2}}{2\sigma_{x}^{2}}-\frac{{\left(y-{\bar{y}}_{t}\right)}^{2}}{2\sigma_{y}^{2}}\right),$$

where $\left({\bar{x}}_{t},{\bar{y}}_{t}\right)$ is the box center and the kernel scales are tied to the box size, $\sigma_{x}=w_{t}/2$ and $\sigma_{y}=h_{t}/2$, with $w_{t}=x_{2}^{t}-x_{1}^{t}$ and $h_{t}=y_{2}^{t}-y_{1}^{t}$ measured in pixels at the evaluation resolution H×W. The kernel therefore adapts to the extent of each evidence box instead of using a fixed spatial scale; varying the tying coefficient κ in $\sigma_{x}=\kappa \;w_{t}$, $\sigma_{y}=\kappa \;h_{t}$ over {0.25,0.5,0.75} changes P-AUROC by at most 0.005 (Section 4.9). For A1 actions that retrieve a full-view observation without a region index, the VLM also emits a coarse spatial descriptor (a quadrant or grid cell). If no spatial descriptor can be parsed, that step contributes only to the image-level score. The final map is the element-wise maximum over all step-level maps:

(21) $$S\left(x,y\right)=\max_{t\in \{1,\ldots ,T-1\}}S_{t}\left(x,y\right).$$

When necessary, *S* is bilinearly interpolated to the evaluation resolution. The corresponding map-derived image score is $\max_{x,y}S\left(x,y\right)$. For the reported I-AUROC and S-AUROC results, the final verbalized anomaly probability and its fallback are used as specified in Section 4.1. We stress that this evidence-derived map is a coarse localization signal rather than a dense segmentation: it aggregates the regions that the policy actually inspected, weighted by confidence. Its validity is assessed against dedicated dense-localization baselines through P-AUROC and AUPRO in Section 4.2, and the sensitivity of the kernel construction is reported in Section 4.9.

### 3.6. Stage 1: Perception-Activated Supervised Fine-Tuning

Before RL optimization, PA-SFT provides an initialization that aligns perception with the structured action format, consistent with the SFT-before-RL recipe of Vision-R1 [44], VLM-R^3^ [17], and IAD-R1 [12]. For each training instance $\left(x,y^{*}\right)$, a teacher policy $\pi_{\mathrm{teach}}$ uses ground-truth annotations to generate a trajectory $\tau^{*}=\left(a_{1}^{*},e_{1}^{*},\ldots ,a_{T-1}^{*},e_{T-1}^{*},A4,{\hat{y}}^{*}\right)$. The heuristic applies the following cases in order:

(22) $$\pi_{\mathrm{teach}}\left(a_{t}|s_{t},y^{*}\right)=\left\{\begin{array}{cc} A1(v_{\mathrm{oblique}},m_{\mathrm{geo}}) & \mathrm{if}\;y^{*}\in K_{\mathrm{geo}}\;\mathrm{and}\;t=1,\\ A2(r_{\mathrm{defect}}) & \mathrm{if}\;y^{*}\in K_{\mathrm{tex}}\;\mathrm{and}\;t=1,\\ A4 & \mathrm{if}\;c_{t-1}\geq \tau_{h}\;\mathrm{or}\;t=B,\\ A3(r_{\mathrm{defect}}) & \mathrm{if}\;c_{t-1}<\tau_{c},\\ A2(r_{\mathrm{defect}}) & \mathrm{otherwise}, \end{array}\right.$$

where $K_{\mathrm{geo}}$ and $K_{\mathrm{tex}}$ are the geometry-dependent and texture defect subsets of K, $m_{\mathrm{geo}}\in \left\{\mathrm{pseudo}\text{-}3D,\mathrm{point}\;\mathrm{cloud}\right\}$, presented through the corresponding geometric renderings of Section 3.4, $\tau_{c}=0.7$ is the comparison threshold, and $\tau_{h}=0.9$ is the early-termination threshold.

The SFT loss is the standard next-token cross-entropy over teacher trajectories:

(23) $$L_{\mathrm{SFT}}=-\sum_{\left(\tau^{*},x\right)\in D_{\mathrm{train}}}\sum_{k=1}^{|\tau^{*}|}\log p_{\theta }\;\left(w_{k}^{*}|w_{<k}^{*},\;x\right),$$

where $w_{k}^{*}$ is the *k*-th token in the serialized teacher trajectory. We apply low-rank adaptation (LoRA) adapters to the language layers (query, key, value, and output projections) with rank 64 and scaling factor 128, keeping the visual encoder frozen. Training uses 3 epochs with a cosine schedule (peak learning rate $2\times 10^{-4}$, warmup ratio 0.03), a per-device batch size of 4, and 8 gradient-accumulation steps.

### 3.7. Stage 2: GRPO-Based Policy Optimization

Starting from the PA-SFT checkpoint, GRPO optimizes the observation-selection policy. GRPO is used in its on-policy form: at each iteration, the behavior policy $\pi_{\theta_{\mathrm{old}}}$ is synchronized with the current $\pi_{\theta }$, *G* complete Algorithm 1 episodes are rolled out per training instance, and a single gradient step is taken on the objective of Equation (25); the importance ratio is computed over the generated tokens of the structured output. Algorithm 2 summarizes the procedure.
**Algorithm 2** GRPO Training for ActiveInspect**Require:** PA-SFT checkpoint $\pi_{\theta_{0}}$, training set $D_{\mathrm{train}}$, group size *G*, budget *B*, iterations $N_{\mathrm{iter}}$ 1: $\pi_{\mathrm{ref}}\leftarrow \pi_{\theta_{0}}$▹ Freeze reference policy 2: **for** $n=1,\ldots ,N_{\mathrm{iter}}$ **do** 3:       Sample mini-batch $B\subset D_{\mathrm{train}}$, |B|=16 4:       **for** each instance $(x,y^{*})\in B$ **do** 5:             **for** i=1,…,G **do**▹G=8 trajectories per instance 6:                  $\tau_{i}∼\pi_{\theta }\left(\cdot |x,B\right)$ via Algorithm 1 7:                  $R\left(\tau_{i}\right)\leftarrow$ ComputeReward$\left(\tau_{i},y^{*}\right)$▹ Equation (30) 8:             **end for** 9:             $\mu_{R}\leftarrow \frac{1}{G}\sum_{i=1}^{G}R\left(\tau_{i}\right)$;    $\sigma_{R}\leftarrow \sqrt{\frac{1}{G}\sum_{i=1}^{G}{\left(R\left(\tau_{i}\right)-\mu_{R}\right)}^{2}}$10:             $A_{i}\leftarrow \left(R\left(\tau_{i}\right)-\mu_{R}\right)/\left(\sigma_{R}+\epsilon \right)$    ∀i∈{1,…,G}11:       **end for**12:       $\theta \leftarrow \theta -\eta \;\nabla_{\theta }L_{\mathrm{GRPO}}$▹ Equation (25)13: **end for**14: **return** $\pi_{\theta }$

**Group-relative advantage.** For each instance, G=8 trajectories are sampled. The advantage of trajectory $\tau_{i}$ is:

(24) $$A_{i}=\frac{R\left(\tau_{i}\right)-\mu_{R}}{\sigma_{R}+\epsilon },\;\mu_{R}=\frac{1}{G}\sum_{j=1}^{G}R\left(\tau_{j}\right),\;\sigma_{R}=\sqrt{\frac{1}{G}\sum_{j=1}^{G}{\left(R\left(\tau_{j}\right)-\mu_{R}\right)}^{2}}.$$

**Clipped surrogate objective.** The policy is updated via:

(25) $$L_{\mathrm{GRPO}}=-E_{\tau }\;\left[\sum_{t=1}^{T}\min\;\left(\rho_{t}A_{\tau },\;\mathrm{clip}\left(\rho_{t},1-\epsilon_{c},1+\epsilon_{c}\right)\;A_{\tau }\right)\right]+\lambda_{\mathrm{KL}}\;D_{\mathrm{KL}}\;\left(\pi_{\theta }∥\pi_{\mathrm{ref}}\right),$$

where $\rho_{t}=\pi_{\theta }\left(a_{t}|s_{t}\right)/\pi_{\theta_{\mathrm{old}}}\left(a_{t}|s_{t}\right)$ and $\epsilon_{c}=0.2$; an automatic termination at budget exhaustion emits no tokens and contributes no term to the sum.

**KL regularization variants.** We compare three KL-regularization strategies, each based on the per-token unbiased estimator relative to the reference policy [41]; DAPO [47] and PAPO [48] motivate the removal and the perception-aware weighting, respectively:

(26) $$d_{k}=\frac{\pi_{\mathrm{ref}}\left(w_{k}|w_{<k}\right)}{\pi_{\theta }\left(w_{k}|w_{<k}\right)}-\log\frac{\pi_{\mathrm{ref}}\left(w_{k}|w_{<k}\right)}{\pi_{\theta }\left(w_{k}|w_{<k}\right)}-1\;\geq \;0.$$

Let $N_{\mathrm{tok}}$ denote the number of generated tokens of the structured output over which the per-token estimator is averaged. The three variants are:

(27) $$D_{\mathrm{KL}}^{\mathrm{std}}=\frac{1}{N_{\mathrm{tok}}}\sum_{k=1}^{N_{\mathrm{tok}}}d_{k},\;\lambda_{\mathrm{KL}}=0.01,$$

(28) $$D_{\mathrm{KL}}^{\mathrm{none}}=0,\;\lambda_{\mathrm{KL}}=0,$$

(29) $$D_{\mathrm{KL}}^{\mathrm{percept}}=\frac{1}{N_{\mathrm{tok}}}\sum_{k=1}^{N_{\mathrm{tok}}}\left\{\begin{array}{cc} \lambda_{\mathrm{KL}}^{\mathrm{act}}\;d_{k} & w_{k}\in W_{\mathrm{action}},\\ \lambda_{\mathrm{KL}}^{\mathrm{rsn}}\;d_{k} & w_{k}\in W_{\mathrm{reason}}, \end{array}\right.\;\lambda_{\mathrm{KL}}^{\mathrm{act}}=0.005,\;\;\lambda_{\mathrm{KL}}^{\mathrm{rsn}}=0.02,$$

where $W_{\mathrm{action}}$ and $W_{\mathrm{reason}}$ denote the action-field and reasoning-field token positions of the structured output. The perception-aware variant regularizes reasoning tokens more strongly than action tokens, permitting greater adaptation of the selection policy while constraining drift in the generated analysis. A quantitative comparison of the three variants, together with the training convergence curves, is provided in Section 4.5.

We train for 500 iterations with a constant learning rate of $5\times 10^{-6}$ and mini-batches comprising 16 instances with 8 trajectories per instance. The training budget is B=5: this value covers the initial observation plus up to four evidence acquisitions and exceeds the 95th percentile of teacher-trajectory lengths in Section 3.6, while larger budgets increase GRPO sampling cost linearly and Section 4.8 shows accuracy saturating near B=3–4; inference-time budgets are varied in Section 4.8. Training uses bfloat16 mixed precision, gradient checkpointing, and eight NVIDIA A100 80 GB GPUs (NVIDIA Corporation, Santa Clara, CA, USA).

### 3.8. Reward Design

The trajectory reward combines terminal objectives, step-level shaping terms, and a length penalty:

(30) $$R\left(\tau \right)=R_{\mathrm{cls}}+\alpha \;R_{\mathrm{loc}}+\beta \;R_{\mathrm{fmt}}+\sum_{t=1}^{T-1}\left(\gamma \;R_{\mathrm{info}}\left(t\right)+\delta \;R_{\mathrm{cons}}\left(t\right)\right)-\lambda \;T.$$

The step-level terms are summed into the trajectory return; the sum runs over the evidence-acquiring steps t=1,…,T−1 of Equation (6), since the terminating step generates no evidence entry. Advantage estimation remains trajectory-level and group-relative (Section 3.7); thus, these terms shape the return without introducing a separate per-step advantage estimator.

**Classification reward.**

(31) $$R_{\mathrm{cls}}=\left\{\begin{array}{cc} +1.0 & \mathrm{correct}\;\mathrm{classification}\;\mathrm{and}\;\mathrm{correct}\;\mathrm{defect}\;\mathrm{type},\\ +0.5 & \mathrm{correct}\;\mathrm{normal}/\mathrm{anomalous}\;\mathrm{but}\;\mathrm{wrong}\;\mathrm{defect}\;\mathrm{type},\\ -1.0 & \mathrm{incorrect}\;\mathrm{normal}/\mathrm{anomalous}\;\mathrm{classification}. \end{array}\right.$$

**Localization reward.**

(32) $$R_{\mathrm{loc}}=\mathrm{IoU}\left({\hat{r}}_{\mathrm{defect}},\;r_{\mathrm{gt}}\right)=\frac{|{\hat{r}}_{\mathrm{defect}}\cap r_{\mathrm{gt}}|}{|{\hat{r}}_{\mathrm{defect}}\cup r_{\mathrm{gt}}|},$$

where the intersection over union (IoU) compares the predicted defect box ${\hat{r}}_{\mathrm{defect}}$ of the final verdict with the ground-truth region $r_{\mathrm{gt}}$. Set to 0 when no spatial annotation is available (α←0).

**Format reward.**

(33) $$R_{\mathrm{fmt}}=\left\{\begin{array}{cc} +0.2 & \mathrm{all}\;\mathrm{required}\;\mathrm{fields}\;\mathrm{present}\;\mathrm{and}\;\mathrm{parseable},\\ -0.5 & \mathrm{any}\;\mathrm{required}\;\mathrm{field}\;\mathrm{missing}\;\mathrm{or}\;\mathrm{malformed}. \end{array}\right.$$

**Information gain reward.**

(34) $$R_{\mathrm{info}}\left(t\right)=\max\;\left(0,\;c_{t}^{\left(\mathrm{gt}\right)}-c_{t-1}^{\left(\mathrm{gt}\right)}\right),$$

where $c_{t}^{\left(\mathrm{gt}\right)}$ is the VLM’s confidence in the ground-truth label at step *t*, with $c_{0}^{\left(\mathrm{gt}\right)}$ parsed from the global-scan output. The max(0,·) operation avoids assigning a negative reward when an exploratory observation temporarily lowers confidence. Because this term depends on the ground-truth label, it is used only during training.

**Cross-view consistency reward.** For all cross-view evidence pairs up to step *t*:

(35) $$C_{t}=\left\{\left(i,j\right):r_{i}=r_{j},\;\left(v_{i},m_{i}\right)\neq \left(v_{j},m_{j}\right),\;i,j\leq t\right\},$$

(36) $$R_{\mathrm{cons}}\left(t\right)=\left\{\begin{array}{cc} \frac{1}{|C_{t}|}\sum_{\left(i,j\right)\in C_{t}}1\left[y_{i}=y_{j}\right] & \mathrm{if}\;|C_{t}|>0,\\ 0 & \mathrm{otherwise}. \end{array}\right.$$

**Efficiency penalty.** The linear term λT penalizes the number of observations consumed (T=|τ|, Equation (6)) and therefore favors early termination when the accumulated evidence is sufficient.

The reward weights are selected via grid search on the Real-IAD D^3^ validation split (Section 4.1):

(37) α=0.3,β=0.1,γ=0.15,δ=0.1,λ=0.05.

The sensitivity of the results to the efficiency coefficient λ is analyzed in Section 4.9.

## 4. Experiments

### 4.1. Experimental Setup

We evaluate ActiveInspect on the six benchmarks summarized in Table 3. Real-IAD D^3^ [33] serves as the primary testbed. Each sample contains five viewpoints represented by RGB, pseudo-3D, and 3D point-cloud data, giving a complete pool of 5×3=15 observations. Real-IAD [31] evaluates selection among five fixed RGB viewpoints with sample-level aggregation, whereas MVTec 3D-AD [30] evaluates switching between RGB and 3D evidence. MVTec-AD [1], VisA [57], and MMAD [10] provide one image per sample; for these datasets, A1 is disabled so that the remaining action, memory, and training components can be evaluated in a single-image setting. We use the official train/test splits and reserve 10% of the Real-IAD D^3^ training set for reward-weight selection.

The six benchmarks are complementary in scale and role. Real-IAD D^3^ spans 20 object categories with the largest per-sample pool (15 observations across 3 physical sensing principles) and therefore exercises every action family; Real-IAD covers 30 categories but restricts the pool to 5 RGB viewpoints, isolating view selection from modality selection; and MVTec 3D-AD contributes 10 categories with exactly 1 RGB–3D pair per sample, so that the decision reduces to whether geometric evidence is worth its cost. The three single-image datasets carry no selection decision at all and instead probe the digital actions, the memory, and the reasoning quality of the backbone, with MMAD adding a question-answering protocol that stresses evidence integration. Together, the collection decouples the contributions of view selection, modality selection, digital refinement, and reasoning, which the ablations of Section 4.4 exploit.

All experiments use Qwen2.5-VL-7B-Instruct (Alibaba Cloud, Hangzhou, China) as the VLM backbone. PA-SFT (Section 3.6) uses one teacher trajectory per training sample from Real-IAD D^3^, Real-IAD, and MVTec 3D-AD, for approximately 18,000 trajectories in total. Trajectories from the single-image datasets are restricted to A2, A3, and A4. GRPO follows the configuration in Section 3.7, with perception-aware KL regularization as the default. Hyperparameters are shared across datasets; apart from the reward-weight search in Section 3.8, no dataset-specific tuning is performed.

The comparisons include three types of baselines. Passive single- and multi-sensor detectors comprise PatchCore [3], SimpleNet [4], M3DM [34], a BTF-style classical-feature baseline [35], Multi-Flow [38], and the D^3^M reference for Real-IAD D^3^ [33]. VLM- and RL-based single-image methods include AnomalyGPT [8], AnomalyR1 [11], IAD-R1 [12], and AgentIAD [13]. Because the official AgentIAD implementation is designed for single-image MMAD evaluation, we also implement an *AgentIAD-style* baseline for the multi-sensor datasets. It uses the same backbone and reproduces the perceptive-zoomer and comparative-retriever tools, with web search disabled. This baseline can refine the default RGB image but cannot retrieve another view or modality. Finally, three observation-selection baselines isolate the contribution of a learned adaptive policy: *Random 3-obs* samples three observations uniformly, *Heuristic 3-obs* uses top-down RGB, one oblique surface-normal rendering, and one point-cloud curvature rendering, and *Exhaustive D^3^* processes all fifteen observations as an oracle reference.

**Information access of the compared methods.** Because the baselines were designed for different input regimes, Table 4 makes their information access explicit. The passive detectors consume their full designated observation set for every test sample and are trained on the complete normal set of each dataset, whereas the VLM policies observe at most *B* inputs chosen at test time and share a single training configuration across datasets. Random 3-obs, Heuristic 3-obs, and the exhaustive scan draw on exactly the same pool, backbone, and prompts as ActiveInspect, and therefore isolate the value of learned, sample-adaptive selection under matched access. In the converse direction, the modality-restricted variants of Section 4.6 evaluate ActiveInspect itself under the reduced access conditions of the passive baselines, so the comparison is anchored from both sides.

**Statistical reporting.** Unless noted otherwise, every ActiveInspect result is the mean over three independent runs of the full PA-SFT + GRPO pipeline (seeds 13, 42, and 87), reported as mean ± one standard deviation. The deterministic embedding-based baselines are run once under their official protocols, and published numbers are used where available. Significance is assessed with a two-sided Wilcoxon signed-rank test over per-category I-AUROC (or S-AUROC) between ActiveInspect and the strongest budget-constrained baseline of each table; the markers ^†^ and ^‡^ denote p<0.05 and p<0.01, respectively. The association tests used in the policy analysis are specified in Section 4.8.

**Hardware and cost.** PA-SFT and GRPO run on eight A100 80 GB GPUs with bfloat16 mixed precision; PA-SFT takes approximately 6 wall-clock hours and GRPO approximately 60 h (about 530 A100-hours in total), with trajectory sampling dominating the GRPO cost. All inference and efficiency measurements use a single A100 80 GB with batch size 1, greedy decoding, and FlashAttention-2 (v2.8.3); the peak inference memory is 21.4 GB. The framework is implemented in Python 3.11 with PyTorch 2.12.0 and Hugging Face Transformers 5.9.0. Reported per-sample times include geometric-rendering preprocessing (0.28 s per observation on average) and exclude disk I/O. Batching four samples raises throughput by about 3.1× without changing observation counts; batch size 1 is reported because it matches sequential in-line deployment.

The following protocol and reporting conventions are used throughout.

**Evaluation metrics.** The area under the receiver operating characteristic curve (AUROC) summarizes the true-positive/false-positive trade-off over all decision thresholds, with AUROC = 1 denoting perfect ranking. We report image-level AUROC (I-AUROC) for binary image classification, pixel-level AUROC (P-AUROC) for anomaly localization, and sample-level AUROC (S-AUROC) for Real-IAD after aggregating the views of each physical sample. The F1 score is the harmonic mean of precision *P* and recall *R*:

(38) $$F1=\frac{2PR}{P+R},$$

computed at the threshold that maximizes it on the test set. The area under the per-region overlap curve (AUPRO) evaluates localization quality by integrating the per-region true-positive rate up to a fixed false-positive rate, following the MVTec 3D-AD convention [30]. Geo-AUROC and Tex-AUROC denote I-AUROC computed over the subsets of samples whose ground-truth defect type is geometry-dependent (dent, warp, concavity, edge chip, crack, pit) or texture-related (scratch, stain, discoloration, contamination), respectively. For MMAD, we report average accuracy in percent, following the benchmark convention [10]. Unless stated otherwise, all AUROC and F1 values are reported on a 0–1 scale with three decimals; in table headers, AUROC is shortened to AUC and average observations to “Obs.” for compactness.

**Score derivation and reporting conventions.** The continuous score required for AUROC is the verbalized anomaly probability produced during verdict generation (Section 3.5). If this value cannot be parsed, which occurs in fewer than 0.3% of test cases, we use the normalized confidence-weighted consensus margin from Equation (19). An *observation* denotes one visual input event supplied to the VLM. The initial RGB image counts as one observation; each executed action A1, A2, or A3 adds one; A4 adds none. The average observation counts are computed on the complete test set, so the single-pass methods (1.0) and the 15-observation exhaustive reference are measured on the same scale. In the tables, **bold** identifies the best budget-constrained result. Exhaustive or oracle rows are retained as upper reference bounds and are not bolded; ties are bolded jointly.

### 4.2. Comparison with State-of-the-Art Methods

Table 5 presents the primary results on Real-IAD D^3^. Relative to the passive D^3^M reference, ActiveInspect increases I-AUROC from 0.890 to 0.906 and F1 from 0.834 to 0.852 while reducing the mean observation count from 3.0 to 2.7. The improvement is stable across seeds (I-AUROC 0.906±0.003) and significant relative to D^3^M under a per-category Wilcoxon signed-rank test (p=0.002, n=20 categories). The AgentIAD-style baseline uses 3.4 observations yet obtains 0.858 I-AUROC, 0.048 below ActiveInspect, which indicates that digital refinement of the default image does not replace access to complementary views and modalities in this setting. With 2.7 observations on average, ActiveInspect reaches 0.906 I-AUROC compared with 0.908 for exhaustive processing of all 15 observations. Thus, it preserves 99.8% of the exhaustive I-AUROC while using 18% of the exhaustive observation count.

Table 6 evaluates view selection on Real-IAD. ActiveInspect-adaptive achieves 0.962 S-AUROC, compared with 0.959 for Multi-Flow, and also improves P-AUROC and F1 while using 2.6 rather than 5.0 views per sample. The margin over Multi-Flow is significant over the 30 categories (p=0.004). Its S-AUROC is within 0.001 of the exhaustive five-view reference; under this protocol, the learned subset therefore captures nearly all of the benefit available from the full pool.

Table 7 reports results for the RGB–3D setting of MVTec 3D-AD. ActiveInspect-RGBD obtains the highest I-AUROC (0.951) and AUPRO (0.968) and matches the BTF-style baseline in P-AUROC (0.993). With only 10 categories, the Wilcoxon test against the BTF-style baseline reaches p=0.037, so the I-AUROC margin is significant at the 0.05 level but not at the 0.01 level. The mean count of 2.4 observations arises because the policy often stops after RGB for visually unambiguous samples, but retrieves 3D evidence or a digital refinement when the initial image is insufficient.

### 4.3. Single-Image Compatibility

Table 8 evaluates ActiveInspect with A1 disabled, leaving A2, A3, and A4 available. This setting isolates the structured digital actions, evidence memory, and two-stage training on standard single-image benchmarks. ActiveInspect-single gives the strongest result within the VLM-RL group in all reported columns, including an average MMAD accuracy of 83.04%, compared with 82.21% for AgentIAD and 80.43% for IAD-R1. The improvement over AgentIAD, which provides a similar set of digital operations, suggests that representing tool use as a structured policy and optimizing it with inspection-specific rewards is beneficial. Fully supervised detectors such as PatchCore nevertheless retain higher raw I-AUROC on MVTec-AD and VisA. The VLM-RL setting instead provides semantic reasoning and a common interface that can be extended to multi-sensor selection. On Real-IAD D^3^, the single-image variant is 0.060 I-AUROC below the complete model (Table 9), providing an estimate of the contribution associated with access to additional views and modalities.

### 4.4. Ablation Study

**Figure 2:**
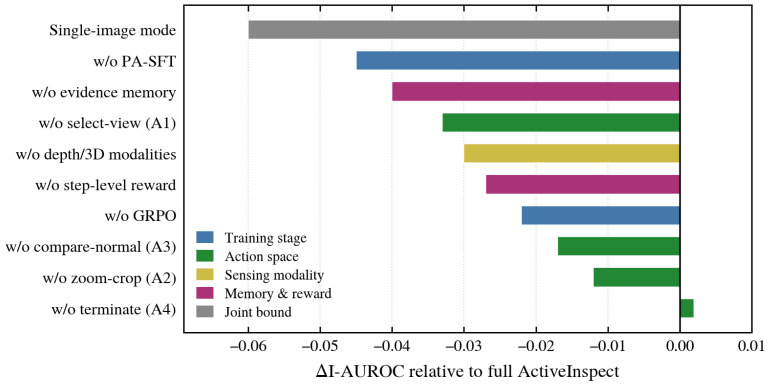
Component-wise change in I-AUROC on Real-IAD D^3^ relative to the complete ActiveInspect model. Components are grouped by function; the single-image setting jointly disables view- and modality-selection components.

Table 9 reports the component ablations in Real-IAD D^3^, and Figure 2 summarizes the corresponding changes in I-AUROC.

The training procedure is most sensitive to the PA-SFT initialization. Removing PA-SFT reduces I-AUROC by 0.045 and increases the mean observation count from 2.7 to 3.6, consistent with a less stable exploration of an unaligned action representation. Using the PA-SFT policy without GRPO reduces I-AUROC by 0.022. Reward-based optimization therefore improves on direct imitation of the teacher trajectories.

Among the action components, disabling view/modality selection (A1) produces a 0.033 reduction in I-AUROC, while disabling zoom-crop (A2) reduces it by 0.012. The removal of depth and 3D observations causes a 0.030 reduction, concentrated in Geo-AUROC. These differences show that complementary geometric observations contribute more than digital enlargement alone on this dataset. Disabling termination (A4) changes I-AUROC by only +0.002 but increases the mean observation count from 2.7 to the full budget of 5.0; therefore, learned termination reduces the observation count by 46% without material loss in accuracy.

Removing structured evidence memory reduces I-AUROC by 0.040; therefore, multiple observations are most useful when they are integrated in a consistent representation. Removing the rewards for information-gain and consistency in view reduces I-AUROC by 0.027 and increases the length of the trajectory. The single-image configuration is 0.060 below the complete model, reflecting the combined contribution of view- and modality-selection components.

Figure 2 groups the deltas of Table 9 by function and makes three patterns visible at a glance. First, the two training-related bars are the largest accuracy contributors, with PA-SFT (−0.045) exceeding GRPO (−0.022), so representation alignment matters more than reward optimization alone. Second, within the action group the ordering A1 > depth/3D > A3 > A2 shows that access to complementary sensors outweighs digital refinement of the recorded image. Third, the A4 bar is the only near-zero accuracy bar, yet removing it almost doubles the observation count; the figure thereby separates the components that drive accuracy (training, memory, sensor access) from the single component that drives efficiency (learned termination).

### 4.5. Training Dynamics and KL-Regularization Comparison

**Figure 3:**
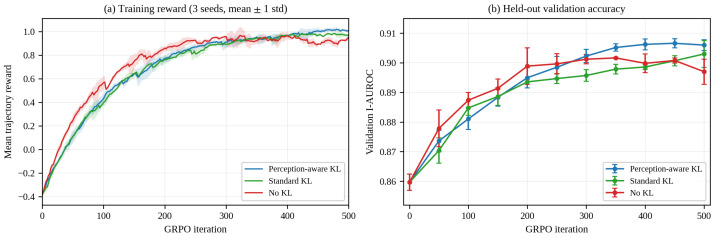
GRPO training dynamics on Real-IAD D^3^. (**a**) Mean trajectory reward versus iteration for the three KL-regularization strategies (mean of three seeds; shaded: ±1 std). (**b**) Validation I-AUROC on the held-out split, evaluated every 50 iterations.

Figure 3 reports the optimization dynamics of the three KL strategies of Section 3.7, each trained with three seeds. The mean trajectory reward rises steeply within the first 150 iterations and plateaus between iterations 350 and 400 for the perception-aware and standard variants. Removing KL regularization accelerates early progress but destabilizes training after roughly 300 iterations, with visible reward oscillation and a decline of the validation I-AUROC from its peak of 0.901 to 0.897 at iteration 500; the format-violation rate of the unregularized policy also triples. Table 10 summarizes the end-of-training outcomes: the perception-aware variant attains the best accuracy and the lowest format-violation rate, supporting the design choice of regularizing reasoning tokens more strongly than action tokens. The narrow inter-seed bands in Figure 3a indicate that the reported differences are not artifacts of a particular initialization.

### 4.6. Defect-Type and Modality Analysis

**Figure 4:**
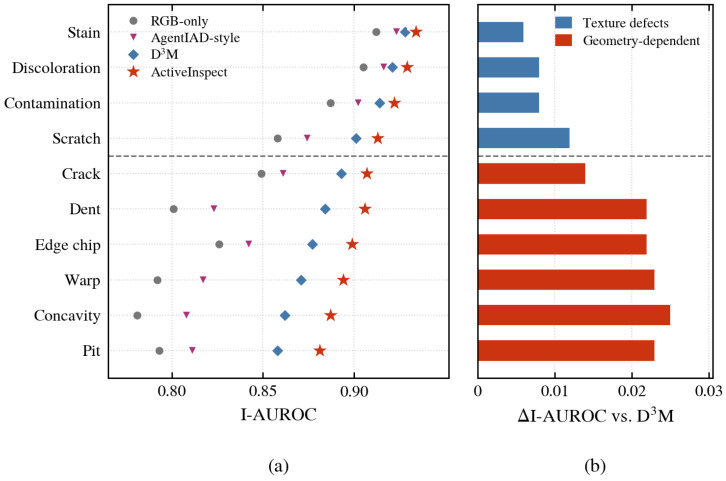
Defect-type analysis on Real-IAD D^3^. (**a**) Per-defect I-AUROC of the RGB-only baseline, the AgentIAD-style baseline, the passive D^3^M reference, and ActiveInspect; the dashed line separates texture defects (top) from geometry-dependent defects (bottom). (**b**) I-AUROC gain of ActiveInspect over D^3^M for each defect type.

Figure 4 decomposes I-AUROC by defect type on Real-IAD D^3^. Improvements over D^3^M are 0.006–0.012 for texture defects but 0.014–0.025 for geometry-dependent defects. The greatest gains occur for concavities (+0.025), warping (+0.023), and pits (+0.023), for which top-down RGB provides limited shape information. Compared with the RGB-only baseline, several geometry-related categories improve by more than 0.08 AUROC; concavity, for example, increases from 0.781 to 0.887. ActiveInspect also exceeds the passive three-modality D^3^M reference for every reported defect type. The result indicates that performance depends not only on making geometric modalities available but also on selecting them according to the sample and suspected region.

Table 11 further examines the complementarity of modality. RGB alone performs best among the single modalities for texture defects (Tex-AUC 0.871) but has the lowest Geo-AUC (0.818). pseudo-3D shows the opposite tendency, with Geo-AUC 0.846 and Tex-AUC 0.809; for Dent/Warp, it reaches 0.861 compared with 0.801 for RGB. Pairwise combinations recover much of the performance of the full pool, and RGB+pseudo-3D is the strongest pair at 0.897 I-AUROC. Using all three modalities yields the highest value in every reported accuracy column. The mean observation count rises from 1.8 for RGB alone to 2.7 for the full pool; the policy therefore uses the larger set selectively rather than evaluating every available modality.

### 4.7. Efficiency Analysis

**Figure 5:**
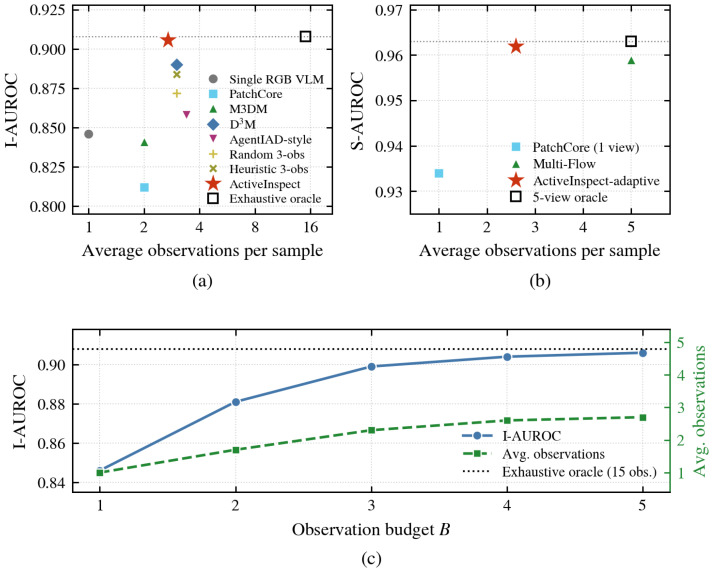
Accuracy–efficiency analysis. (**a**) I-AUROC versus average observations per sample on Real-IAD D^3^ (logarithmic horizontal axis); methods toward the upper left achieve a better trade-off, and the dotted line marks the exhaustive oracle. (**b**) S-AUROC versus average observations on Real-IAD. (**c**) Effect of the inference-time observation budget *B* on Real-IAD D^3^ for a policy trained with B=5.

Figure 5a,b compares accuracy with average observation count, and Table 12 reports runtime and normalized cost under the same implementation. All timing entries follow the measurement protocol of Section 4.1: a single A100 80 GB, batch size 1, bfloat16, greedy decoding, preprocessing included, and disk I/O excluded, with a peak memory of 21.4 GB. On Real-IAD D^3^, ActiveInspect uses 2.7 observations, compared with 3.0 for both fixed-budget baselines, while increasing I-AUROC from 0.872 for Random 3-obs and 0.884 for Heuristic 3-obs to 0.906. The improvement therefore reflects sample-dependent selection rather than a fixed three-observation recipe. Relative to exhaustive processing, ActiveInspect reduces runtime from 16.8 s to 3.2 s per sample (5.3×) while decreasing I-AUROC by 0.002. Under the same pool-based implementation, the AgentIAD-style baseline also requires more observations and time while obtaining lower accuracy. Real-IAD shows a similar accuracy–observation relationship: ActiveInspect-adaptive attains a higher S-AUROC than Multi-Flow with approximately half as many views. The efficiency coefficient λ of Equation (30) controls this trade-off directly; its sensitivity is quantified in Section 4.9.

### 4.8. Policy Behavior and View-Budget Analysis

Figure 5c varies the inference-time budget *B* for a policy trained with B=5. I-AUROC increases from 0.846 at B=1, equivalent to the single-RGB setting, to 0.899 at B=3. Beyond this point it approaches saturation: increasing the budget from four to five observations adds 0.002 I-AUROC, and the realized mean remains 2.7 because the policy can end before reaching the limit. A budget of B=3 retains 99.2% of the full-model I-AUROC while lowering the worst-case trajectory length. Across the three seeds, the value at every budget varies by at most ±0.003 I-AUROC, so the shape of the curve is stable.

**Figure 6:**
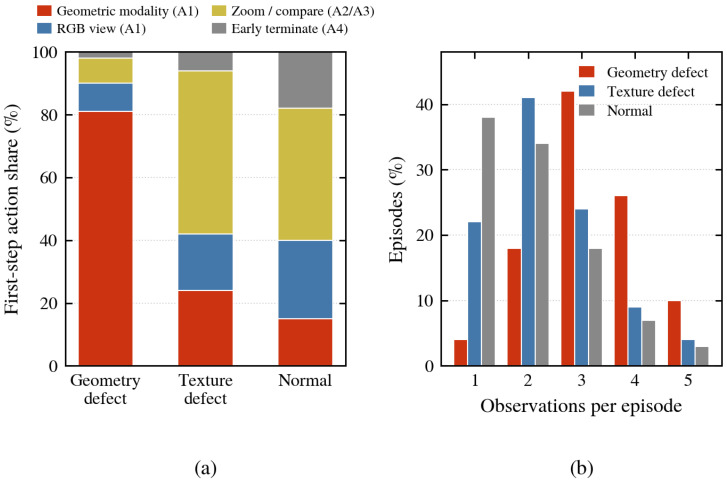
Learned observation-selection behavior on Real-IAD D^3^. (**a**) First post-initial action by sample category. (**b**) Episode-length distribution by sample category. Geometry-related defects select geometric modalities earlier and use longer trajectories, whereas texture-defect and normal samples tend to terminate earlier.

The trajectory statistics in Figure 6 show a clear association between the suspected defect type and the selected evidence. For geometry-dependent defects, the first post-initial action selects a geometric modality, presented as a surface-normal, curvature, or point-cloud rendering, in 81% of trajectories, compared with 24% for texture defects. In contrast, 63% of texture-defect episodes end within two observations, whereas geometry-defect episodes use 3.2 observations on average. These associations are statistically supported. A chi-square test of independence between the sample class (geometry defect, texture defect, normal) and the first post-initial action group (geometric modality, additional RGB view, digital A2/A3, terminate) yields $\chi^{2}\left(6\right)=412.7$, p<0.001, with Cramér’s V=0.39, and a two-sided Mann–Whitney U test confirms that geometry-defect episodes are longer than texture-defect episodes ($U=1.84\times 10^{5}$, p<0.001, rank-biserial r=0.41). A3 is selected mainly when the step confidence lies between 0.4 and 0.7 and is uncommon after high-confidence observations. The learned behavior follows the broad structure of the PA-SFT teacher but is not identical to it; for example, the GRPO policy selects a second geometric view for some ambiguous warp cases, an action absent from the teacher heuristic.

### 4.9. Sensitivity Analyses

**Figure 7:**
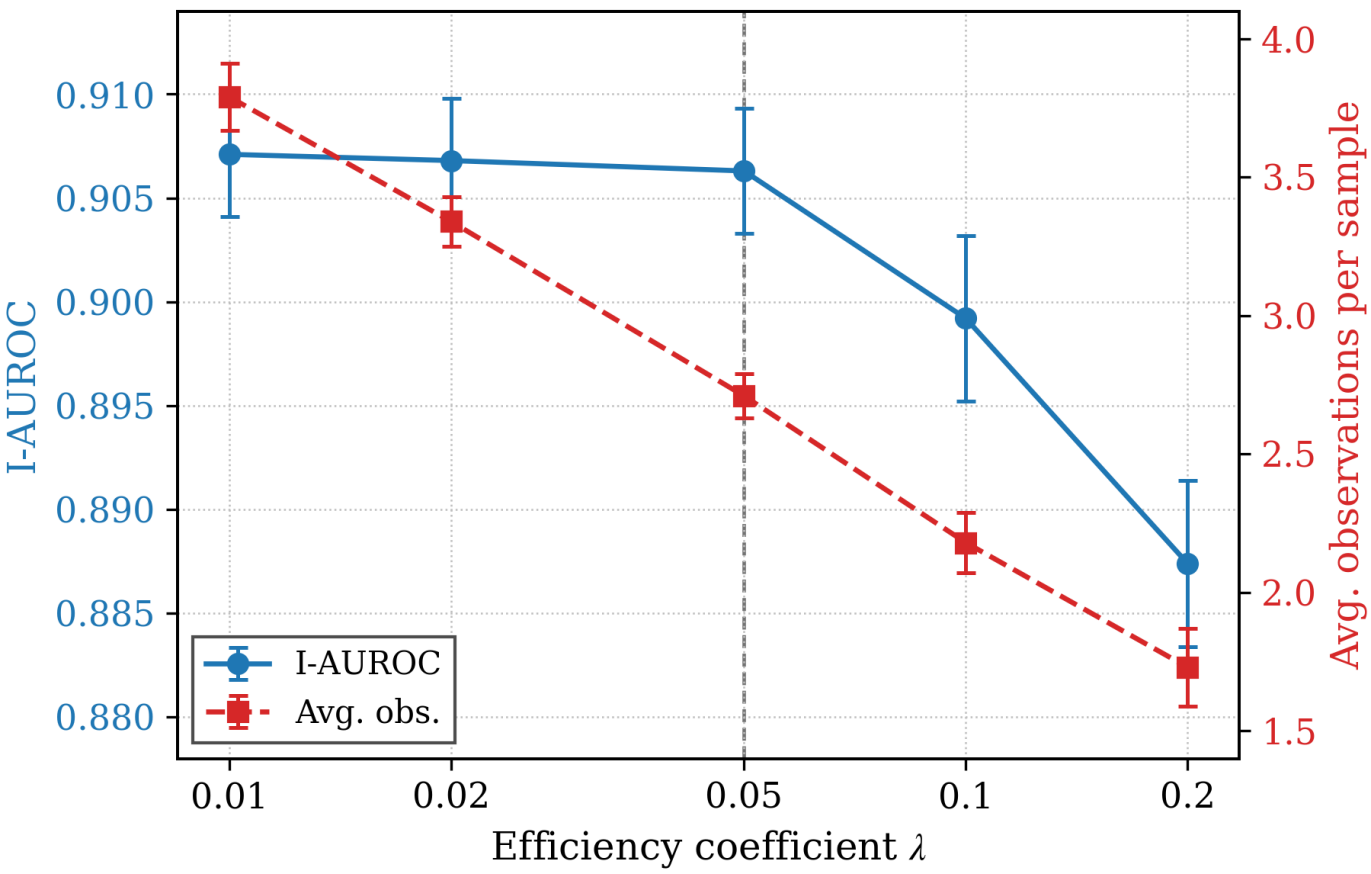
Sensitivity of ActiveInspect to the efficiency coefficient λ on Real-IAD D^3^. Left axis: I-AUROC; right axis: mean observation count. Error bars: ±1 std over three seeds; the dashed line marks the default λ=0.05.

**Efficiency coefficient λ.** Figure 7 sweeps λ∈{0.01,0.02,0.05,0.1,0.2} in Equation (30) with all other hyperparameters fixed, retraining the GRPO stage for each value. Accuracy is flat to within 0.001 I-AUROC for λ≤0.05, while the mean observation count falls from 3.8 to 2.7; beyond λ=0.05 the policy truncates evidence gathering prematurely, and accuracy degrades to 0.887 at λ=0.2 with only 1.7 observations. The default λ=0.05 therefore sits at the knee of the accuracy–efficiency curve, and the reported advantages are not the artifact of a finely tuned penalty: any value in [0.02,0.05] reproduces the main results within one standard deviation.

**Zoom-grid granularity.** Table 13 varies the A2 grid of Section 3.3. The 2×2 grid loses 0.009 I-AUROC: its cells average one quarter of the image, so small defects are diluted before the resize step. The 4×4 grid adds only 0.001 I-AUROC while increasing the mean observation count and enlarging the A2 parameter space to N≤20. The defect-size statistics of the primary benchmark explain this behavior: the median ground-truth defect box covers 2.1% of the image area (interquartile range 0.8–5.6%), against 11.1% for one 3×3 cell, so a single crop at the default granularity already restores sufficient effective resolution for typical defects, and the sub-cell tail is handled by the free-form VLM proposals rather than by a finer grid.

**Score-map kernel scale.** For the pixel-level map of Equation (21), scaling both kernel widths by κ∈{0.25,0.5,0.75} yields P-AUROC values of 0.941, 0.946, and 0.942 on Real-IAD D^3^ and AUPRO values of 0.961, 0.968, and 0.964 on MVTec 3D-AD. The box-tied default κ=0.5 is used throughout; the localization scores are thus not the product of kernel tuning.

### 4.10. Robustness to Calibration Errors and Sensor Perturbations

**Figure 8:**
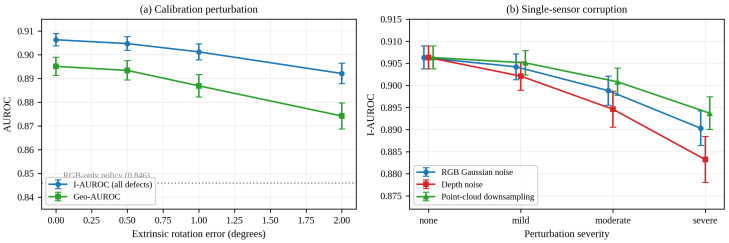
Robustness of ActiveInspect on Real-IAD D^3^. (**a**) Test-time extrinsic calibration error applied before re-rendering the geometric modalities; the dotted line marks the RGB-only policy. (**b**) Single-sensor corruption at increasing severity: RGB Gaussian noise (σ=0/8/16/32), depth noise (σ=0/0.5/1/2 mm), and point-cloud downsampling (100/75/50/25% of points). Error bars: ±1 std over three seeds.

Because the surface-normal, curvature, and point-cloud renderings of Section 3.4 depend on the camera model, we perturb the calibration at test time and re-render all geometric observations without retraining the policy. Figure 8a applies extrinsic rotation errors of up to $2^{\circ }$; focal-length errors of up to ±2% change I-AUROC by at most 0.004. I-AUROC decreases gracefully from 0.906 to 0.892 at $2^{\circ }$, and Geo-AUROC from 0.895 to 0.874, both remaining well above the RGB-only policy (0.846): miscalibration degrades but does not invalidate the geometric evidence.

Figure 8b corrupts individual sensors: additive Gaussian noise on RGB (σ up to 32 on the 0–255 scale), depth noise before rendering (σ up to 2 mm), and random point-cloud downsampling to 25% of the points. The largest degradation is 0.023 I-AUROC under severe depth noise. Trajectory inspection reveals a compensating behavior: under severe depth noise the policy selects point-cloud renderings 1.7× more often than in the clean setting, indicating that adaptive selection itself provides a degree of robustness to single-sensor degradation that a fixed observation schedule cannot offer. A systematic analysis of the residual failure modes is given in Section 5.4.

## 5. Discussion

### 5.1. Active Evidence Selection as a Learnable Inspection Strategy

The results support the treatment of observation selection as part of the inspection model rather than as a fixed preprocessing choice. Conventional anomaly detectors optimize representations and scoring functions for a predetermined input, while most VLM-RL systems optimize reasoning over an image that has already been supplied. ActiveInspect also learns which evidence available should enter the decision process. This distinction is relevant because the informative observation is sample dependent: a texture defect may be resolved from the initial RGB image, whereas a subtle deformation may require an oblique geometric rendering. GRPO is therefore used not only to improve the final answer but also to optimize the sequence of evidence on which that answer is based.

### 5.2. Digital Refinement Versus Complementary Sensor Evidence

Digital refinement and multi-sensor selection address different sources of uncertainty. A crop can improve the effective resolution of a recorded region, but it cannot introduce shape cues that were not present in the original RGB acquisition. Surface-normal, curvature, and point-cloud renderings provide complementary geometric measurements and can therefore resolve cases with weak appearance contrast. This interpretation is consistent with the ablation results: eliminating A2 reduces I-AUROC by 0.012, whereas removing A1 or the depth/3D modalities reduces it by 0.033 and 0.030, respectively. The comparison does not imply that zooming is not important; rather, it shows that digital refinement and sensor complementarity make distinct contributions, the latter being more consequential for the geometry-heavy benchmark Real-IAD D^3^, echoing the conclusion of AgentIAD [13] that digital tools alone are bounded by the information content of the captured image.

### 5.3. Defect Classes That Benefit from Multi-Sensor Selection

The defect-level analysis in Section 4.6 shows that the largest gains occur for defects that alter surface geometry, including dents, warping, concavities, edge chips, and pits. Their appearance in RGB can depend strongly on illumination and viewpoint, whereas normal and curvature renderings encode the local shape more directly. Improvements are smaller for stains and discoloration because the initial RGB image already contains much of the discriminative information. The trajectory analysis is consistent with this division: geometry-related samples trigger geometric modalities more frequently and produce longer inspection sequences, whereas many texture defects terminate after one additional observation or fewer. These findings suggest that adaptive selection is most useful when the candidate modalities encode genuinely different physical properties, in line with the sensor-complementarity observations reported for MulSen-AD [32].

### 5.4. Failure Modes

A manual review of the Real-IAD D^3^ test errors of the best seed (214 misclassified samples) identifies four recurring failure modes. (i) Sub-resolution micro-defects (38% of errors): defects covering less than 0.3% of the image area are missed both by the grid cells and by the free-form proposals, and no modality switch can recover detail that the region-proposal stage never isolates. (ii) Specular and transparent surfaces (27%): reflections corrupt the photometric-stereo normals, producing spurious geometric evidence; these cases dominate the false positives and connect directly to the depth-noise degradation of Section 4.10. (iii) Cross-modality conflict (19%): RGB and geometric evidence disagree and the verdict follows the wrong side; the consensus of Equation (19) reduces but does not eliminate such cases. (iv) Ambiguous ground truth (16%): borderline samples near the annotation threshold, for which the policy typically exhausts the budget before terminating. Modes (i) and (ii) suggest concrete extensions, namely higher-resolution tiling for micro-defects and polarization- or reflectance-aware rendering for specular parts.

### 5.5. Practical Deployment Considerations

The term “active” in this study refers to selection from pre-acquired multi-view and multi-modal pools. The policy does not yet move a camera, switch a physical sensor, or trigger online acquisition. Consequently, the reported observation count measures VLM input events rather than the physical sensing cost. An action of A1 would incur sensor and motion overhead in a deployed system, while actions of A2 and A3 are primarily computational operations. Observation count should therefore be interpreted as a controlled measure of evidence consumption, not as a complete measure of time, energy, or hardware cost.

However, the pool-based protocol provides a reproducible way to compare selection strategies while keeping the available evidence fixed. A physical implementation would require additional measurements, including VLM calls, sensor acquisition and switching time, preprocessing latency, motion-planning overhead, and end-to-end cycle time, quantities for which the view-planning literature provides established cost models [50,51]. It would also introduce failure modes absent from the current evaluation, such as occlusion after repositioning, calibration drift, and failed acquisitions; the calibration-perturbation analysis of Section 4.10 is a first step toward quantifying the second of these. In the present implementation, learned termination reduces the mean to 2.7 observations and B=3 retains more than 99% of the full-model I-AUROC. Further reductions in latency can be obtained through policy distillation or by using the VLM policy to schedule smaller defect-specific perception modules.

## 6. Limitations

Several limitations remain. First, view and modality selection is evaluated on pre-acquired pools rather than through closed-loop robotic sensing. A physical system would add motion planning, calibration, sensor-switching delays, and acquisition failures. Second, the current observation metric assigns an equal weight to A1, A2, and A3 even though their deployment costs differ. Future evaluations should report action-specific costs, VLM calls, sensor and preprocessing time, and end-to-end latency in addition to observation count, for which the cost models of the active-sensing literature [52,53] offer a principled starting point. Third, the sensor suite is limited to RGB, photometric-stereo pseudo-3D, and point clouds; richer suites such as the laser and lock-in infrared channels of MulSen-AD [32], as well as thermal, X-ray, ultrasonic, and hyperspectral inspection, may require different encodings and policies. Fourth, the 7B VLM remains more computationally demanding than compact discriminative detectors, although early termination and model distillation may reduce this gap. Fifth, comparisons with single-image baselines involve different information-access conditions. The common counting protocol, the information-access summary of Table 4, the matched-pool selection baselines, and the single-image ablation partially address this issue, but standardized active-inspection benchmarks are needed for a more direct comparison.

## 7. Conclusions

We presented ActiveInspect, a GRPO-optimized framework for budget-constrained selection of multi-view and multi-modal evidence in industrial defect detection. The method integrates view/modality selection, digital refinement, normal-reference comparison, structured evidence memory, and learned termination within a single VLM policy initialized by PA-SFT. Across six benchmarks, the framework improves the accuracy–observation trade-off, with the largest gains on defects whose diagnosis depends on surface geometry. In Real-IAD D^3^, it achieves 0.906 I-AUROC using 2.7 observations on average, compared to 0.908 for exhaustive processing of 15 observations. The present results establish the value of learning which evidence to inspect in a controlled pool-based setting. Extending the policy to closed-loop sensor control with explicit per-action cost models, enabling online adaptation and continual learning on evolving production lines and distilling the policy for edge deployment in resource-constrained inspection stations are the main directions for future work.

## Figures and Tables

**Table 1:** Capability comparison of representative methods. ✓ = supported, ✗ = not supported. “Obs.” = average observations per sample, as reported by the original work or measured under the protocol of Section 4.1. Bold marks our method.

	VLM Reason.	RL Train.	Zoom Crop	Normal Ref.	View Sel.	Modal. Sel.	Evid. Mem.	Obs.
PatchCore [3]	✗	✗	✗	✗	✗	✗	Bank	1.0
SimpleNet [4]	✗	✗	✗	✗	✗	✗	✗	1.0
M3DM [34]	✗	✗	✗	✗	✗	✓	Bank	2.0
D^3^M [33]	✗	✗	✗	✗	✗	✓	Bank	3.0
Multi-Flow [38]	✗	✗	✗	✗	Fixed	✗	Flow	5.0
AnomalyGPT [8]	✓	✗	Weak	Opt.	✗	✗	✗	1.0
AnomalyR1 [11]	✓	GRPO	✗	✗	✗	✗	✗	1.0
IAD-R1 [12]	✓	SC-GRPO	✗	✗	✗	✗	✗	1.0
AgentIAD [13]	✓	Agent	✓	✓	✗	✗	Tool	3.4
**ActiveInspect**	✓	PA-SFT +GRPO	✓	✓	✓	✓	Struct.	2.7

**Table 3:** Dataset roles and evaluation dimensions. Metric abbreviations are defined in the evaluation-protocol paragraph of this section.

Dataset	Observation Type	Main Metrics	Active Component Tested
Real-IAD D^3^ [33]	RGB + pseudo-3D + 3D	I-AUROC, P-AUROC, F1	View/modality selection, memory
Real-IAD [31]	5 fixed RGB views	S-AUROC, P-AUROC, F1	View selection, early stop
MVTec 3D-AD [30]	RGB + 3D scan	I-AUROC, P-AUROC, AUPRO	Modality switching
MVTec-AD [1]	RGB	I-AUROC, P-AUROC	Backbone (no active view)
VisA [57]	RGB	I-AUROC, P-AUROC	Backbone (no active view)
MMAD [10]	Visual question answering on industrial images	Average accuracy	Reasoning & formatting

**Table 4:** Information access of the compared methods on Real-IAD D^3^. “Full pool” denotes all 15 pre-acquired observations. In the last column, ✓ = the observation set is selected adaptively for each sample, ✗ = the observation set follows a fixed schedule.

Method	Training Signal	Test-Time Access	Obs./Sample	Adaptive
PatchCore [3]	full normal set	RGB + 3D, fixed schedule	2.0	✗
M3DM [34]	full normal set	RGB + 3D, fixed schedule	2.0	✗
D^3^M [33]	full normal set	RGB + pseudo-3D + 3D, fixed	3.0	✗
AgentIAD-style [13]	same backbone, tool SFT	default RGB + digital tools	3.4	digital only
Random 3-obs	same backbone as ActiveInspect	full pool, random subset	3.0	✗
Heuristic 3-obs	same backbone as ActiveInspect	full pool, fixed recipe	3.0	✗
ActiveInspect	PA-SFT + GRPO	full pool, policy-selected, ≤B	2.7	✓
Exhaust. D^3^ (oracle)	same backbone as ActiveInspect	full pool, all observations	15.0	✗

**Table 5:** Main results on Real-IAD D^3^. Arrows indicate the preferred direction (↑: higher is better; ↓: lower is better). Bold: best among budget-constrained methods; the exhaustive scan is an upper-bound reference. ActiveInspect rows: mean ± std over three seeds; ^‡^ marks p<0.01 versus D^3^M under a per-category Wilcoxon signed-rank test.

Method	Setting	I-AUROC ↑	P-AUROC ↑	F1 ↑	Obs. ↓
PatchCore	RGB + 3D passive	0.812	0.905	0.781	2.0
M3DM	RGB + 3D passive	0.841	0.922	0.802	2.0
D^3^M [33]	RGB + pseudo-3D + 3D passive	0.890	0.937	0.834	3.0
AgentIAD-style	RGB + digital tools	0.858	0.906	0.791	3.4
**ActiveInspect-D^3^**	active multi-sensor	**0.906** ± 0.003 ^‡^	**0.946** ± 0.002	**0.852** ± 0.004	2.7 ± 0.1
Exhaust. D^3^ (oracle)	all 15 observations	0.908	0.948	0.855	15.0

**Table 6:** Main results on Real-IAD (multi-view selection). All metrics on a 0–1 scale. Arrows indicate the preferred direction (↑: higher is better; ↓: lower is better). Bold: best among budget-constrained methods; the 5-view oracle is an upper-bound reference. ActiveInspect row: mean ± std over three seeds; ^‡^: p<0.01 versus Multi-Flow (Wilcoxon over 30 categories).

Method	S-AUROC ↑	P-AUROC ↑	F1 ↑	Obs. ↓
PatchCore (1 view)	0.934	0.942	0.837	1.0
Multi-Flow [38]	0.959	0.959	0.858	5.0
**ActiveInspect-adaptive**	**0.962** ± 0.002 ^‡^	**0.961** ± 0.002	**0.863** ± 0.003	2.6 ± 0.1
Exhaust. 5-view (oracle)	0.963	0.962	0.865	5.0

**Table 7:** Main results on MVTec 3D-AD (RGB–3D compatibility). Arrows indicate the preferred direction (↑: higher is better; ↓: lower is better). Bold: best among budget-constrained methods; ties are bolded jointly. ActiveInspect row: mean ± std over three seeds; ^†^: p<0.05 versus the BTF-style baseline (Wilcoxon over 10 categories).

Method	I-AUROC ↑	P-AUROC ↑	AUPRO ↑	Obs. ↓
PatchCore (RGB)	0.875	0.961	0.914	1.0
M3DM [34]	0.936	0.987	0.956	2.0
BTF-style [35]	0.944	**0.993**	0.962	2.0
**ActiveInspect-RGBD**	**0.951** ± 0.003 ^†^	**0.993** ± 0.001	**0.968** ± 0.002	2.4 ± 0.1

**Table 8:** Single-image compatibility (active view/modality selection disabled). Bold indicates the best result within each method category.

Method	Setting	MVTec-AD		VisA		MMAD
		I-AUC	P-AUC	I-AUC	P-AUC	Avg. Acc. (%)
*Traditional (full normal training)*						
PatchCore [3]	full normal	**0.990**	**0.981**	**0.954**	**0.972**	–
SimpleNet [4]	full normal	0.988	0.979	0.951	0.969	–
*Zero-/few-shot*						
WinCLIP [5]	zero/few	0.918	0.857	0.784	0.858	–
AnomalyCLIP [6]	zero-shot	0.943	0.918	0.866	0.931	–
PromptAD [7]	few-shot	**0.955**	**0.936**	**0.902**	**0.948**	–
*VLM-RL*						
AnomalyGPT [8]	1-normal-shot	0.941	0.952	0.887	0.921	72.80
AnomalyR1 [11]	VLM-RL	0.947	0.956	0.902	0.934	76.96
IAD-R1 [12]	PA-SFT + SC-GRPO	0.961	0.963	0.918	0.945	80.43
AgentIAD [13]	tool-agent	0.965	0.966	0.925	0.951	82.21
**ActiveInspect-single**	PA-SFT + GRPO	**0.968**	**0.968**	**0.929**	**0.954**	**83.04**

**Table 9:** Core ablation on Real-IAD D^3^. Arrows indicate the preferred direction (↑: higher is better; ↓: lower is better). Bold marks the complete ActiveInspect model, which is the reference configuration for the ΔI-AUC column.

Variant	I-AUC ↑	P-AUC ↑	F1 ↑	Geo-AUC ↑	Avg. Obs. ↓	ΔI-AUC
ActiveInspect full	**0.906**	**0.946**	**0.852**	**0.895**	2.7	–
w/o PA-SFT	0.861	0.912	0.801	0.842	3.6	−0.045
w/o GRPO	0.884	0.929	0.826	0.871	3.3	−0.022
w/o select-view A1	0.873	0.923	0.813	0.854	2.5	−0.033
w/o zoom-crop A2	0.894	0.936	0.839	0.883	2.4	−0.012
w/o compare-normal A3	0.889	0.934	0.834	0.876	2.6	−0.017
w/o terminate A4	0.908	0.947	0.854	0.897	5.0	+0.002
w/o depth/3D	0.876	0.919	0.812	0.853	2.3	−0.030
w/o evidence memory	0.866	0.914	0.804	0.849	2.7	−0.040
w/o step-level reward	0.879	0.927	0.821	0.865	3.1	−0.027
single-image mode	0.846	0.895	0.782	0.818	1.8	−0.060

**Table 10:** End-of-training comparison of the KL-regularization variants on Real-IAD D^3^. Arrows indicate the preferred direction (↑: higher is better; ↓: lower is better), and bold marks the best value in each column. Mean ± std over three seeds; the format-violation rate is the fraction of test responses with a missing or malformed required field.

KL Variant	I-AUC ↑	Avg. Obs. ↓	Format Err. (%) ↓	Reward Plateau (Iter.)
Standard ($\lambda_{\mathrm{KL}}=0.01$)	0.903 ± 0.004	2.8 ± 0.1	0.7	≈320
None ($\lambda_{\mathrm{KL}}=0$)	0.901 ± 0.006	2.5 ± 0.1	1.9	≈260, then drift
Perception-aware (default)	**0.906** ± 0.003	2.7 ± 0.1	**0.4**	≈360

**Table 11:** Modality ablation on Real-IAD D^3^: overall, category-level, and representative per-defect metrics. Arrows indicate the preferred direction (↑: higher is better; ↓: lower is better), and bold marks the best value in each column. RGB dominates texture defects, while pseudo-3D and 3D are decisive for geometry-dependent defects.

Modality Setting	I-AUC ↑	P-AUC ↑	Geo-AUC ↑	Tex-AUC ↑	Dent/Warp AUC ↑	Scratch AUC ↑	Avg. Obs. ↓
RGB only	0.846	0.895	0.818	0.871	0.801	0.858	1.8
pseudo-3D only	0.827	0.902	0.846	0.809	0.861	0.832	1.9
Point cloud only	0.804	0.861	0.839	0.771	0.854	0.781	1.8
RGB + point cloud	0.881	0.927	0.868	0.895	0.876	0.887	2.3
RGB + pseudo-3D	0.897	0.941	0.889	0.905	0.904	0.901	2.4
pseudo-3D + point cloud	0.872	0.925	0.881	0.863	0.892	0.866	2.4
All three modalities	**0.906**	**0.946**	**0.895**	**0.917**	**0.911**	**0.913**	2.7

**Table 12:** Efficiency comparison. Arrows indicate the preferred direction (↑: higher is better; ↓: lower is better). Real-IAD D^3^ rows report I-AUROC; Real-IAD rows report S-AUROC. Bold: best among budget-constrained methods per dataset block; see Figure 5 for the corresponding accuracy–observation trade-off. Timing protocol: one A100 80 GB, batch size 1, bfloat16, peak memory 21.4 GB (Section 4.1). “Rel. cost” is the per-sample time normalized by the single-RGB VLM pass (1.1 s) under the same protocol.

Method	Dataset	Obs./Samp. ↓	Time/Samp. ↓	Rel. Cost ↓	AUROC ↑
Single RGB VLM	Real-IAD D^3^	1.0	1.1 s	1.0×	0.846
AgentIAD-style	Real-IAD D^3^	3.4	4.2 s	3.8×	0.858
Random 3-obs	Real-IAD D^3^	3.0	3.6 s	3.3×	0.872
Heuristic 3-obs	Real-IAD D^3^	3.0	3.5 s	3.2×	0.884
ActiveInspect	Real-IAD D^3^	2.7	3.2 s	2.9×	**0.906**
Exhaust. D^3^ (oracle)	Real-IAD D^3^	15.0	16.8 s	15.3×	0.908
Multi-Flow	Real-IAD	5.0	2.8 s	2.5×	0.959
ActiveInspect-adaptive	Real-IAD	2.6	2.9 s	2.6×	**0.962**

**Table 13:** Effect of the A2 grid granularity on Real-IAD D^3^. Arrows indicate the preferred direction (↑: higher is better; ↓: lower is better). Mean ± std over three seeds; *N* is the maximum size of the candidate-region set including the four free-form proposals.

Grid	*N*	I-AUC ↑	P-AUC ↑	Avg. Obs. ↓
2×2	≤8	0.897 ± 0.004	0.938 ± 0.003	2.6 ± 0.1
3×3 (default)	≤13	0.906 ± 0.003	0.946 ± 0.002	2.7 ± 0.1
4×4	≤20	0.907 ± 0.003	0.948 ± 0.002	2.9 ± 0.1

## Data Availability

The data presented in this study are available upon request from the corresponding author. The data are not publicly available due to privacy.
